# SARS-CoV-2 SUD2 and Nsp5 Conspire to Boost Apoptosis of Respiratory Epithelial Cells via an Augmented Interaction with the G-Quadruplex of BclII

**DOI:** 10.1128/mbio.03359-22

**Published:** 2023-02-28

**Authors:** Ying Li, Quanwei Yu, Ridong Huang, Hai Chen, Hequan Ren, Lingling Ma, Yang He, Weimin Li

**Affiliations:** a Department of Respiratory and Critical Care Medicine, Targeted Tracer Research and Development Laboratory, West China Hospital, Sichuan University, Chengdu, China; b Institute of Respiratory Health, Frontiers Science Center for Disease-Related Molecular Network, West China Hospital, Sichuan University, Chengdu, China; c Precision Medicine Research Center, West China Hospital, Sichuan University, Chengdu, China; Duke University School of Medicine

**Keywords:** DNA topology, epithelial cells, gene expression, protein-DNA interactions, protein-protein interactions

## Abstract

The molecular mechanisms underlying how SUD2 recruits other proteins of severe acute respiratory syndrome coronavirus 2 (SARS-CoV-2) to exert its G-quadruplex (G4)-dependent pathogenic function is unknown. Herein, Nsp5 was singled out as a binding partner of the SUD2-N+M domains (SUD2_core_) with high affinity, through the surface located crossing these two domains. Biochemical and fluorescent assays demonstrated that this complex also formed in the nucleus of living host cells. Moreover, the SUD2_core_-Nsp5 complex displayed significantly enhanced selective binding affinity for the G4 structure in the BclII promoter than did SUD2_core_ alone. This increased stability exhibited by the tertiary complex was rationalized by AlphaFold2 and molecular dynamics analysis. In line with these molecular interactions, downregulation of BclII and subsequent augmented apoptosis of respiratory cells were both observed. These results provide novel information and a new avenue to explore therapeutic strategies targeting SARS-CoV-2.

## INTRODUCTION

The 2019 coronavirus disease (COVID-19) pandemic has been one of the longest-lasting and most impactful pandemics in modern human history, with more than 644 million diagnosed cases and 6.6 million deaths (1, 2). Since its outbreak, the causative agent, severe acute respiratory syndrome coronavirus 2 (SARS-CoV-2), has mutated from Alpha to Omicron, making complete prevention of viral infection and spread of the disease extremely difficult, if not impossible (3, 4). Consequently, the elucidation of the underlying mechanisms of the damage caused by SARS-CoV-2 on host cells is of great importance for finding efficient ways to cure or alleviate its severe effects and to inform strategies for future viral epidemics (5).

Previously, most studies on SARS-CoV-2 proteins focused on the spike and RNA-dependent RNA polymerase proteins, which mediate entry and replication of viruses (6, 7). The pathogenic proteins leading to the severe syndrome remain a separate puzzle (8, 9). Historically, the initially discovered strains of human coronaviruses were identified as only mild pathogens (10–12) until the emergence of SARS-CoV in 2003 (13, 14). Subsequent research revealed a sequence exclusively present in the genome of SARS-CoV encoding a special domain located at the N terminus of nonstructural protein 3 (Nsp3) of SARS-CoV (amino acid residues 389 to 652), which was originally named the SARS unique domain (referred to as SUD1 in this study) and was found to be responsible for the enhanced viral pathogenicity (15, 16). One of the most intriguing characteristics of SUD1 was its ability to interact with a special noncanonical DNA structure, the G-quadruplex (G4), which might account for its severe pathogenicity (17–19).

The Nsp3 of SARS-CoV-2 also contains a SUD portion (referred to as SUD2 in this study) in Nsp3, which shares about 75% similarity in amino acid residues with SUD1 (20). The high homology of the two prompted us to investigate how the interactions of SUD2 with G4 are related to its pathogenicity. Our initial experiment indicated that some critical G4s did indeed interact with SUD2 with diverse binding affinities *in vitro*, and a similar phenomenon has been reported by another group as well (20). However, the exact mode of interaction (SUD2-G4) that leads to the severe pathogenicity of SARS-CoV-2 is still unclear.

Importantly, most viral proteins exert their functions as part of multiprotein complexes, particularly for many DNA binding proteins (21, 22). The whole genome of SARS-CoV-2 can encode 27 different proteins, including Nsp1 to -16, structural proteins (S, E, M, and N), and accessory proteins (23, 24). Since SUD2 is a part of Nsp3, we further investigated if SUD2 interacted with other Nsps, whether such interactions influenced the G4 binding property of SUD2, and how such interactions affected the host cell’s fate, which is still an untouched theme.

In this study, using a yeast two-hybrid (Y2H) system (25–27), Nsp5, previously named main proteinase (Mpro) or 3-chymotrypsin-like proteinase (3CL_pro_), was singled out among the Nsps as the only previously unknown partner of interaction for SUD2-N+M (named SUD2_core_ in this study) (28, 29). In host human respiratory cells, both SUD2_core_ and Nsp5 were localized to the cytosol and nucleus, and their interaction in living human cells was confirmed by coimmunoprecipitation (co-IP) and bimolecular fluorescence complementary (BiFC) assays (30, 31). Furthermore, a G4 pulldown assay indicated that Nsp5 strengthened the binding affinity of SUD2_core_ with the *B cell lymphoma 2* (BclII) promoter G4 sequence (*Bcl2G4*). The atomic interaction between SUD2_core_, Nsp5, and *Bcl2G4* was simulated theoretically to infer their crucial structural features. This interaction downregulated the expression of BclII, which in turn led to augmented apoptosis of transfected host cells. Since the induced apoptosis of infected respiratory cells is a hallmark of severe COVID-19 illness (32–35), these results provide a possible explanation for how SUD2_core_, together with its partner Nsp5, lead to severe disease.

## RESULTS

### Screening of SUD2 binding partners in Nsp proteins of SARS-CoV-2.

**FIG 1:**
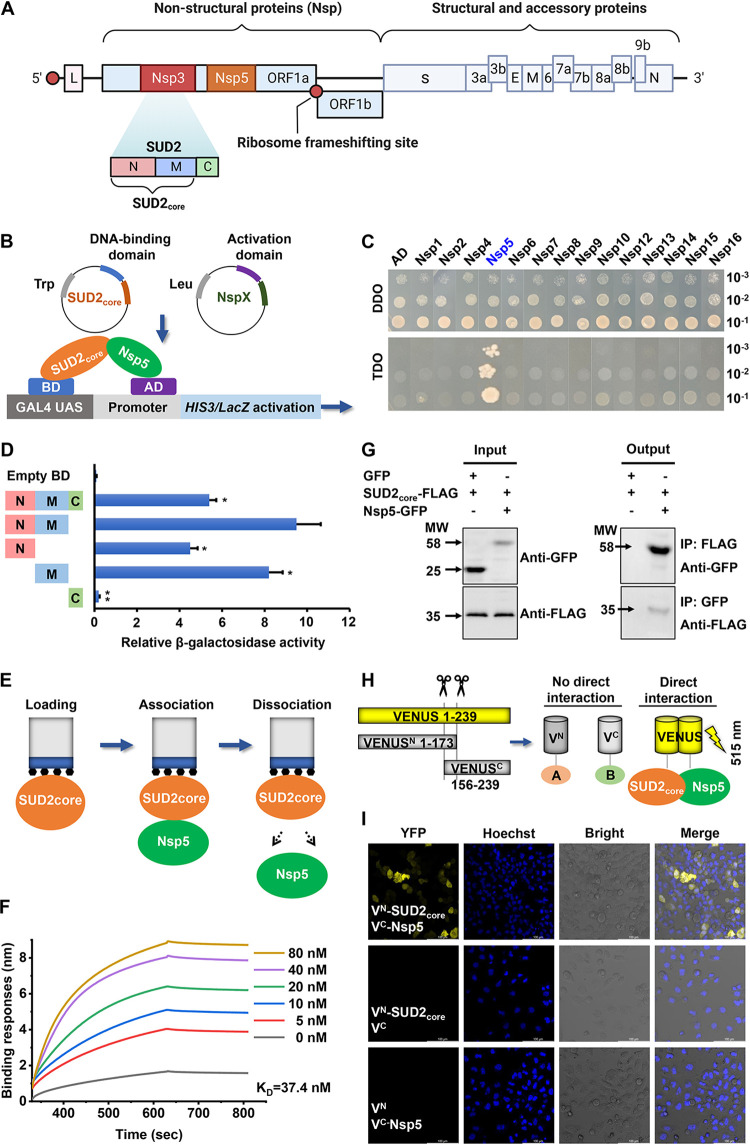
SUD2 interacts with Nsp5 of SARS-CoV-2. (A) Organization of the SARS-CoV-2 genome. (B) Schematic of the Y2H screening assay. (C) Interaction analysis between SUD2_core_ and all Nsps of SARS-CoV-2, determined by growth assay on selective media with series dilutions (10^−1^, 10^−2^, and 10^−3^) of saturated cultures. (D) Detecting the interactions of different SUD2 truncations with Nsp5 in yeast cells by β-galactosidase activity assay. Data represent means ± SEM of triplicate biological results. Statistical significance was calculated by unpaired Student's *t* test. *, *P* < 0.05; **, *P* < 0.01. (E) Schematic of BLI assay. (F) Representative kinetics of the association and dissociation of Nsp5 toward SUD2_core_. (G) Verifying the interaction between SUD2_core_ and Nsp5 by co-IP assay in NHBE cells. (H) Schematic of BiFC experiment. (I) BiFC assays in NHBE cells confirmed the interaction between Nsp5-VENUS^C^ and SUD2_core_-VENUS^N^ under a confocal laser scanning microscope. Scale bars, 100 μm.

SUD2 (from K412 to S743 of SARS-CoV-2 Nsp3) consists of three subdomains: macrodomains II and III and the domain preceding Ubl2 and PL2^pro^ (DPUP) (30, 36), which are also named SUD-N (K412 to E548), SUD-M (I549 to S675), and SUD-C (S676 to S743), respectively (Fig. 1A). In order to investigate the direct interactions of SUD2 with other Nsps of SARS-CoV-2, 14 Nsps were fused to the activation domain (AD^Leu^) to act as 14 distinct preys, and SUD2_core_ (SUD2-N+M [K412 to S675]), due to its *in vitro* stability, was used as the bait fused to the DNA binding domain (BD^Trp^) in the Y2H system (Fig. 1B). Through phenotypic analysis of positive clones growing on synthetically defined (SD) medium lacking histidine, the direct interactions could be efficiently determined, as SUD2_core_ recruits the directly interacting Nsp, thus activating the reporter gene *histidine synthetase 3* (*HIS3*) (37–39).

Successfully transfected yeast clones containing the 14 different AD-Nsps and BD-SUD2_core_ were selected from SD media lacking tryptophan and leucine (SD/-Trp/-Leu [DDO]) (Fig. 1C, upper panel). From the chosen clones, only the one encoding Nsp5 grew well in the SD medium lacking tryptophan, leucine, and histidine (SD/-Trp/-Leu/-His [TDO]) (Fig. 1C, lower panel), indicating that only Nsp5 could directly interact with SUD2_core_. In this clone, the expression of both SUD2_core_ and Nsp5 were verified by appropriate antibodies (see Fig. S1A and B in the supplemental material). The negative-control clones could not activate *HIS3* (Fig. S1C).

10.1128/mbio.03359-22.1 FIG S1 The validation of protein expression of SUD2_core_ and Nsp5 in yeast cells and the growth conditions of negative controls and the truncated clones. (A) Yeast growth assay on SD medium lacking Leu and Trp with serial dilutions (10^−1^, 10^−2^, and 10^−3^) of saturated cultures. (B) The immunoblot of SUD2_core_ and Nsp5 in yeast cells using anti-HA and anti-myc antibodies. (C and D) Yeast growth assay on selective SD (DDO and TDO) medium for negative controls and SUD2 truncations. Download FIG S1, TIF file, 2.6 MB. Copyright © 2023 Li et al. 2023 Li et al. https://creativecommons.org/licenses/by/4.0/ This content is distributed under the terms of the Creative Commons Attribution 4.0 International license.

In order to further examine the contact domains of the SUD2-Nsp5 interaction, the full-length SUD2 (N+M+C) and four truncated versions were generated and scrutinized by the same process. SUD2-N+M+C, SUD2-N+M, SUD2-N, and SUD2-M grew well on TDO media, but SUD2-C did not, suggesting that the full-length and three N- and M-containing truncations interacted with Nsp5 and that SUD2-C did not (Fig. S1D). Next, to quantify the interaction strength of the different truncations, interaction-dependent *LacZ* reporter activities were tested (39). SUD2-N+M showed a significantly enhanced induction effect that was ~2.1-fold higher than the that with the full length of SUD2. Of note, SUD2-N and SUD2-M individually displayed reduced reporter activities (47% and 86%, respectively) compared with SUD2-N+M, and SUD2-C alone lacked this interaction (Fig. 1D). Apart from the enzymatic assay, a high physical affinity (equilibrium disassociation constant [*K_D_*] of 37.4 nM) between SUD2_core_ and Nsp5 was quantitatively determined by biolayer interferometry (BLI) assay, in which the kinetic stability of the SUD2_core_-Nsp5 complex was also indicated via the flat dissociation slopes (Fig. 1E and F).

Consequently, Nsp5 has been clearly distinguished as the only Nsp component of SARS-CoV-2 to interact with SUD2_core_. Moreover, the middle sequence between the N and M domains of SUD2 seems to constitute a major determinant of the Nsp5 interaction, while SUD-C does not interact with Nsp5. According to these results, SUD2_core_ was chosen for the subsequent studies.

### SUD2_core_ and Nsp5 form a complex in respiratory epithelial cells.

After the direct interaction between SUD2_core_ and Nsp5 had been established in yeast cells, the presence of this interaction in normal human bronchial epithelial (NHBE) cells, the first step in SARS-CoV-2 infection (40, 41), was addressed by co-IP assays. The successful expression of green fluorescent protein (GFP), SUD2_core_-FLAG, and Nsp5-GFP was confirmed with input samples (Fig. 1G, left). During anti-FLAG antibody pulldown, Nsp5-GFP was detected with anti-GFP antibody. Conversely, SUD2_core_-FLAG was detected while using anti-GFP as immunoprecipitant (Fig. 1G, right). Nonspecific binding was not detected in the control experiment with GFP empty vector. These results demonstrated that SUD2_core_ and Nsp5 genuinely formed a complex in respiratory epithelial cells.

Regarding intracellular localization, only isolated Nsp3-N (the N terminus of Nsp3) and full-length Nsp5 have been previously reported in HEp-2 cells (42). The subcellular localization of the SUD2_core_-Nsp5 complex in respiratory epithelial cells is as yet unknown. To address this problem, GFP-labeled SUD2_core_ and Nsp5 were separately expressed in NHBE and H1299 cells (Fig. S2A). Through confocal laser scanning microscope (CLSM) visualization, both SUD2_core_ and Nsp5 were found in the cytosol and nucleus of these cells (Fig. S2B).

10.1128/mbio.03359-22.2 FIG S2 SUD2_core_ and Nsp5 localized to the nucleus and cytoplasm of NHBE cells. (A) Immunoblot analysis of SUD2_core_-GFP and Nsp5-GFP in NHBE cells after transfection for 48 h. (B) The subcellular localization of SUD2_core_ and Nsp5 GFP fusions visualized by confocal laser scanning microscope. The cells were transfected with SUD2_core_-GFP and Nsp5-GFP. GFP signal, Hoechst blue stain, and bright-field images of the analyzed and merged images were shown. Scale bars, 100 μm. (C) Immunoblot analysis of YFP fusion proteins in NHBE cells transfected with SUD2_core_-YFP^N^ and Nsp5-YFP^C^ for 48 h. GAPDH was used as the internal control. Download FIG S2, TIF file, 2.4 MB. Copyright © 2023 Li et al. 2023 Li et al. https://creativecommons.org/licenses/by/4.0/ This content is distributed under the terms of the Creative Commons Attribution 4.0 International license.

To validate that the SUD2_core_-Nsp5 complex also formed in cells, BiFC assays were performed (30, 31). The VENUS N-segment fused to SUD2_core_ (SUD2_core_-VENUS^N^) and the VENUS C-segment fused to Nsp5 (Nsp5-VENUS^C^) were cotransfected into NHBE and H1299 cells (Fig. 1H and Fig. S2C). When detected at 515 nm, cells that contained both SUD2_core_-VENUS^N^ and Nsp5-VENUS^C^ proteins yielded a strong yellow fluorescent protein (YFP) signal in both the cytosol and nucleus (Fig. 1I). The negative controls, VENUS^N^+VENUS^C^, SUD2_core_-VENUS^N^+VENUS^C^, and Nsp5-VENUS^C^+VENUS^N^, all failed to display any signal (Fig. 1I). Together, these experiments provided evidence of intracellular SUD2_core_-Nsp5 complex formation in respiratory epithelial cells and showed both nuclear and cytoplasmic subcellular localization.

### The G4 structure in the promoter of BclII has a negative regulatory effect.

Since the SUD2_core_-Nsp5 complex is found in the nucleus, an intriguing question is whether the SUD2_core_-Nsp5 complex can also interact with G4 DNA structures, such as in the cases of individual SUD1 (18, 20, 43, 44), and what the biological consequence of this interaction is. It has been estimated that there are 300,000 sequences in the human genome with the potential to form the G4 structure, and many of them have regulatory effects on gene expression when located in the promoter regions (45, 46). In this study, we chose BclII as the target gene, because its promoter contains a G4 formation sequence and its downregulation is associated with the increased apoptosis of respiratory cells in patients with severe COVID-19 (35).

A 23-nucleotide (nt) sequence (referred as *Bcl2G4* in this study) (Table S1) in the P1 promoter of BclII, about 1,700 nt upstream of the transcriptional start site, has been reported to have the capability to form a G4 structure *in vitro* (47–49). However, the relationship between the G4 structure of this sequence and the reduced expression of BclII in cells was unknown. Thus, we examined the formation of *Bcl2G4* and its effect on the expression of BclII in respiratory epithelial cells.

10.1128/mbio.03359-22.10 TABLE S1 Oligonucleotides used in this study. Download Table S1, DOCX file, 0.02 MB. Copyright © 2023 Li et al. 2023 Li et al. https://creativecommons.org/licenses/by/4.0/ This content is distributed under the terms of the Creative Commons Attribution 4.0 International license.

**FIG 2:**
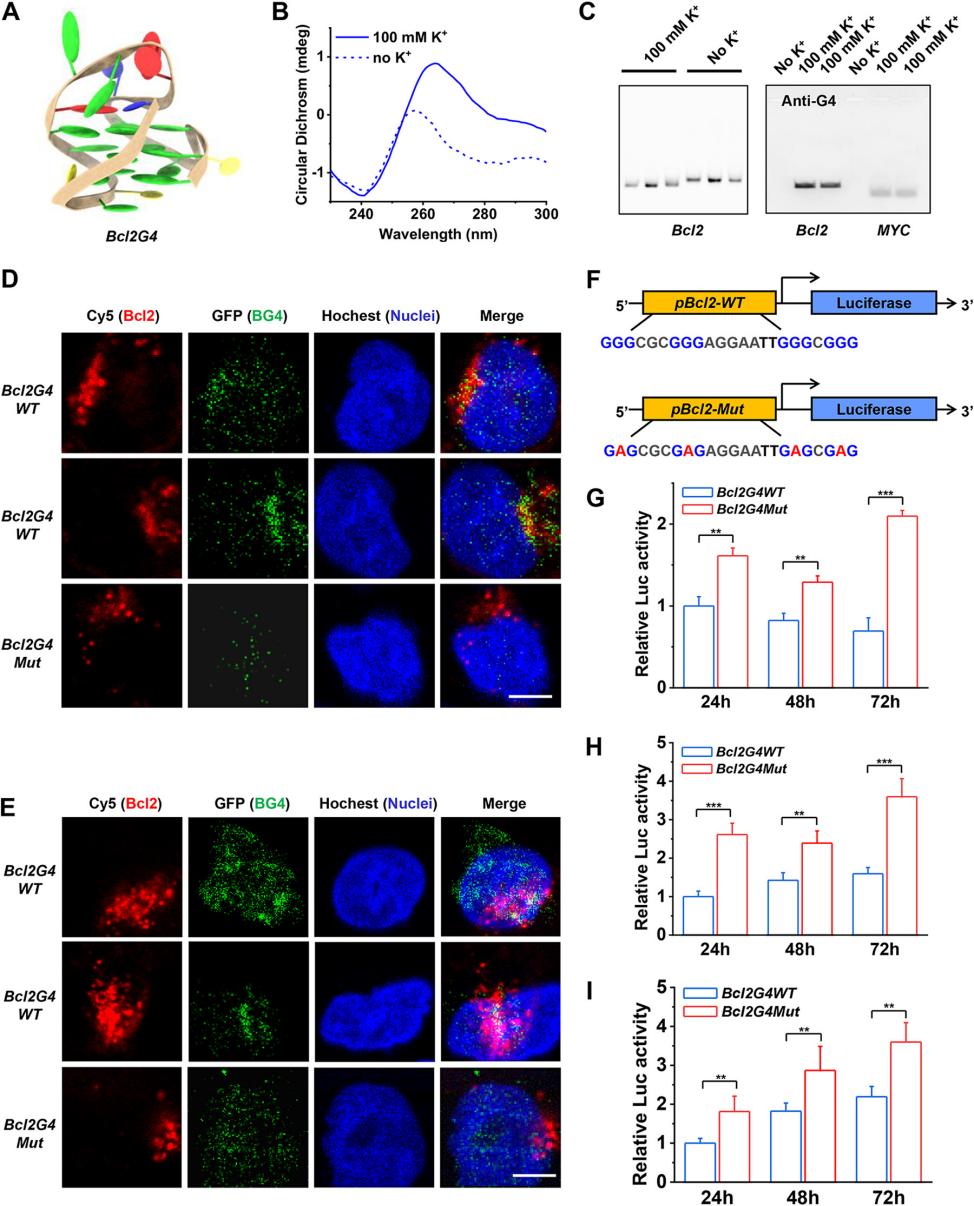
The G-rich region of the BclII promoter forms G4 structure and represses the transcription of BclII. (A) Representation of the parallel topology of *Bcl2G4*. (B) Morphological characteristics of *Bcl2G4* by CD spectrum. (C) Migration analysis of *Bcl2G4* versus single-strand DNA by native PAGE (left), and immunoblot analysis of the *Bcl2G4* and *MYCG4* formation using anti-G4 (BG4) antibody (right). (D and E) Formation of *Bcl2G4* in NHBE (D) and H1299 (E) cells. Colocalization of Cy5-labeled *Bcl2G4* (red) with BG4 antibody (green) was performed by immunofluorescence. Hoechst stain (blue) indicates the nuclei. Scale bars, 10 μm. (F) Schematic presentation of the luciferase reporter constructs. The reporter construct consists of the *Bcl2G4* promoter (WT, wild type; Mut, mutant) controlling the expression of the firefly luciferase (LUC). (G to I) The effect of *Bcl2G4WT* and *Bcl2G4Mut* promoters on LUC expression in NHBE (G), H1299 (H), and A549 (I) cells. The *Renilla* luminescence reporter activity is expressed relative to the control activity. Data represent the means ± SEM of triplicate biological results. Statistical significance was calculated by unpaired Student's *t* test. **, *P* < 0.01; ***, *P* < 0.001.

In the *in vitro* validation, a G4 structure with a predominantly unimolecular parallel topological characteristic formed by *Bcl2G4* in K^+^ buffer (a necessary cationic condition) was indicated by the circular dichroism (CD) spectrum (Fig. 2A and B), which displayed a characteristic positive peak around 264 nm and a negative peak close to 240 nm (50). This was further confirmed in native polyacrylamide gel electrophoresis (PAGE) with either Cy5-labeled or anti-G4 antibody (BG4) immunoblotting (Fig. 2C). *MYCG4*, a well-known G4 structure, was used as a positive control (20). In the intracellular validation, Cy5-labeled *Bcl2G4* DNAs were transfected into NHBE and H1299 cells and detected at 649-nm excitation by CLSM. G4 formation in living cells was then visualized by the BG4 antibody immunofluorescence method (51). The overlap of Cy5 and BG4 signals for the wild-type *Bcl2G4* DNA in living respiratory epithelial cells, appearing as yellow regions, decisively confirmed that *Bcl2G4* can form the G4 structure intracellularly (Fig. 2D and E). In contrast, no G4 structure formation was observed for *Bcl2G4Mut* DNA, in which the corresponding guanines were replaced by adenines in the quadrilateral planes.

Next, the regulatory effect on BclII expression by *Bcl2G4* was investigated. The core region of the BclII promoter containing *Bcl2G4* was constructed in the luciferase (LUC) expression system (Fig. 2F). A dual-luciferase reporter gene activity assay showed that the relative activities of firefly luciferase of *pBcl2G4*-*WT* in all three respiratory epithelial cells were decreased compared to that in the *pBcl2G4*-*Mut* cells, demonstrating that the *Bcl2G4* structure suppressed the transcriptional activity of BclII promoter in host cells (Fig. 2G, H, and I). Based on these experiments, *Bcl2G4* does indeed act as a regulator of BclII transcription.

### SUD2_core_ tightly binds with *Bcl2G4* DNA, and Nsp5 enhances this interaction.

**FIG 3:**
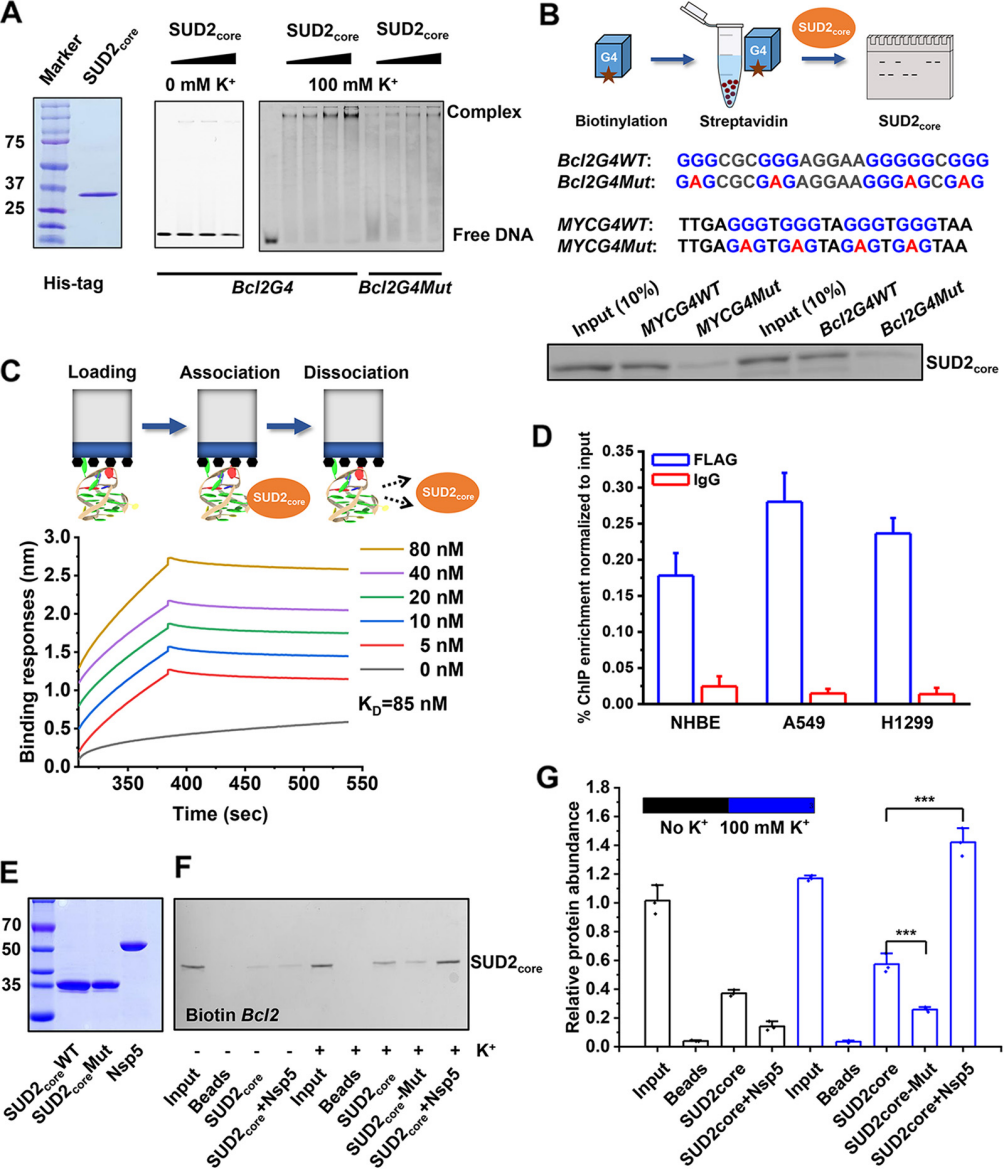
The validation of SUD2_core_-*Bcl2G4* interaction and the enhancement role of Nsp5 for this interaction. (A) Purified SUD2_core_ was shown by Coomassie brilliant blue (CBB) staining (left) and concentration-dependent binding of SUD2_core_ with Cy5-labeled BclII DNA confirmed by native PAGE (right panel). (B) G4 pulldown assay performed for SUD2_core_ with biotin-labeled *Bcl2G4* and *MYCG4* (as a positive control). The amount of retained His-tagged SUD2_core_ with *G4* DNA was detected with anti-His antibody. A 10% dilution of His-SUD2_core_ was used as input. The mutant forms of G4 are named with “Mut.” (C) Binding kinetics of SUD2_core_ with *Bcl2G4* measured by BLI assays. The biotin-labeled G4s were immobilized on the surface of streptavidin (SA) biosensors as indicated in the upper scheme. (D) ChIP-qPCR assays for the confirmation of SUD2_core_-G4 interaction in different cells that constitutively expressed the SUD2_core_ domain. The genomic DNA templates were immunoprecipitated by FLAG antibody or IgG antibody (negative control). (E) Purified Nsp5 and SUD2_core_ detected by CBB staining. (F) Amount of retained His-tagged SUD2_core_ with G4s oligonucleotides was detected with anti-His antibody in a G4 pulldown assay. SUD2_core_-Mut represents for K578A, S582A, and R586A mutants of Nsp3, which abolished the SUD2_core_-*Bcl2G4* interaction based on structural docking. (G) Relative abundance of retained SUD2_core_ corresponding to that in panel F. Data represent the means ± SEM of triplicate biological results. Statistical significance was calculated by unpaired Student's *t* test. ***, *P* < 0.001.

After *Bcl2G4* formation and its role in living cells had been established, its interaction with SUD2 was investigated. Recombinant SUD2_core_ was expressed in Escherichia coli and purified by ion affinity and gel filtration chromatography (Fig. 3A and Fig. S3A to C). An electrophoretic mobility shift assay (EMSA) demonstrated that, under the G4 formation conditions, SUD2_core_ bound tightly to *Bcl2G4* DNA, displaying a lagging band close to the positive pole. In contrast, single-stranded DNA in the non-K^+^ buffer barely bound to SUD2_core_ (Fig. 3A). Additionally, a G4 pulldown assay was also performed (Fig. 3B) to investigate the interactions (20). After incubation with 5′-biotin-labeled *Bcl2G4WT* and then enrichment with streptavidin-coated magnetic beads, SUD2_core_ was detected by immunoblotting with anti-His antibodies, but the *Bcl2G4Mut* sequence displayed much lower band intensity (Fig. 3B). Furthermore, when another G4-forming sequence, *MYCG4*, was used as a positive control, a consistent result was observed (Fig. 3B) (20).

10.1128/mbio.03359-22.3 FIG S3 SUD2_core_ and Nsp5 purification and the detection of stable SUD2_core_-FLAG protein expression induced by lentivirus in NHBE cells. (A) CBB staining of expressed His-tagged SUD2_core_. The proteins were eluted by 200 mM imidazole. (B) Gel filtration profile of the SUD2_core_, showing a sharp peak at 280 nm. (C) SDS gel of the purified SUD2_core_. (D) CBB staining of expressed GST-tagged Nsp5 proteins. (E) The protein levels of SUD2_core_-FLAG in lentivirus-infected cell lines. Download FIG S3, TIF file, 2.8 MB. Copyright © 2023 Li et al. 2023 Li et al. https://creativecommons.org/licenses/by/4.0/ This content is distributed under the terms of the Creative Commons Attribution 4.0 International license.

Quantitatively, in the BLI assay, SUD2_core_ and *Bcl2G4* DNA was found to exhibited a high binding affinity (*K_D_* = 85 nM) (Fig. 3C). By comparison, non-G4 structures in the absence of K^+^ of the *Bcl2G4* sequence showed a 14-fold-higher *K_D_*, 1.22 μM, illustrating the necessity of the G4 structure that mediates the interaction (data not shown). Intracellularly, a chromatin immunoprecipitation quantitative real-time PCR (ChIP-qPCR) assay was employed to verify the SUD2_core_-*Bcl2G4* interaction in NHBE, H1299, and A549 living cells. Constitutive SUD2_core_-FLAG-expressing cell lines were obtained through lentivirus infection (Fig. S3E). In all tested cells, after immunoprecipitation with FLAG antibody and subsequent qPCR, a much higher enrichment of the *Bcl2G4* region was detected, compared with the IgG negative controls (Fig. 3D). Therefore, intracellular complex formation of SUD2_core_ with *Bcl2G4* was confirmed.

The effect of Nsp5 on this SUD2_core_-*Bcl2G4* interaction was then inspected. Following a similar preparation procedure as for His-SUD2_core_, glutathione *S*-transferase (GST)-Nsp5 was obtained (Fig. 3E and Fig. S3D). As can be seen from G4 pulldown assay, the addition of Nsp5 had no impact without G4 formation (in the absence of K^+^) (Fig. 3F). In contrast, in the presence of K^+^, the amount of SUD2_core_ binding with *Bcl2G4* was significantly elevated 6.3-fold higher than without Nsp5 (Fig. 3F and G). Furthermore, in order to examine the effect of Nsp5 on the binding specificity of SUD2_core_, two other G4-forming sequences (*VEGFR2G4* and *KRASG4*) (Table S1) in the promoters of *vascular endothelial growth factor receptor 2* (*VEGFR2*) and *Kirsten Rat sarcoma viral oncogene homolog* (*KRAS*) were employed in comparison experiments (52, 53) (Fig. S4A). Under G4 formation conditions, SUD2_core_ interacted preferentially with *Bcl2G4* and not with *VEGFR2G4* or *KRASG4* (Fig. S4B and C). Upon the addition of Nsp5, the selective binding behavior of SUD2_core_ was not altered.

10.1128/mbio.03359-22.4 FIG S4 G4 pulldown assay showing that SUD2_core_ did not interact with *VEGFR2G4* or *KRASG4*. (A) The CD features of *VEGFR2G4* and *KRASG4* sequences. (B) Amount of retained SUD2_core_ with G4s was detected with anti-His antibody. A 10% dilution of His-SUD2_core_ was used as a positive control. (C) Relative protein abundance of retained SUD2_core_ corresponding to the data shown in panel B. Download FIG S4, TIF file, 2.5 MB. Copyright © 2023 Li et al. 2023 Li et al. https://creativecommons.org/licenses/by/4.0/ This content is distributed under the terms of the Creative Commons Attribution 4.0 International license.

These results demonstrated that Nsp5 enhanced the interaction between SUD2_core_ and *Bcl2G4*, and the tertiary complex of SUD2_core_-Nsp5-*Bcl2G4* was more stable than that of the binary SUD2_core_-*Bcl2G4* alone.

### Inhibition of BclII expression by SUD2_core_ is augmented by Nsp5 in a G4-dependent manner.

**FIG 4:**
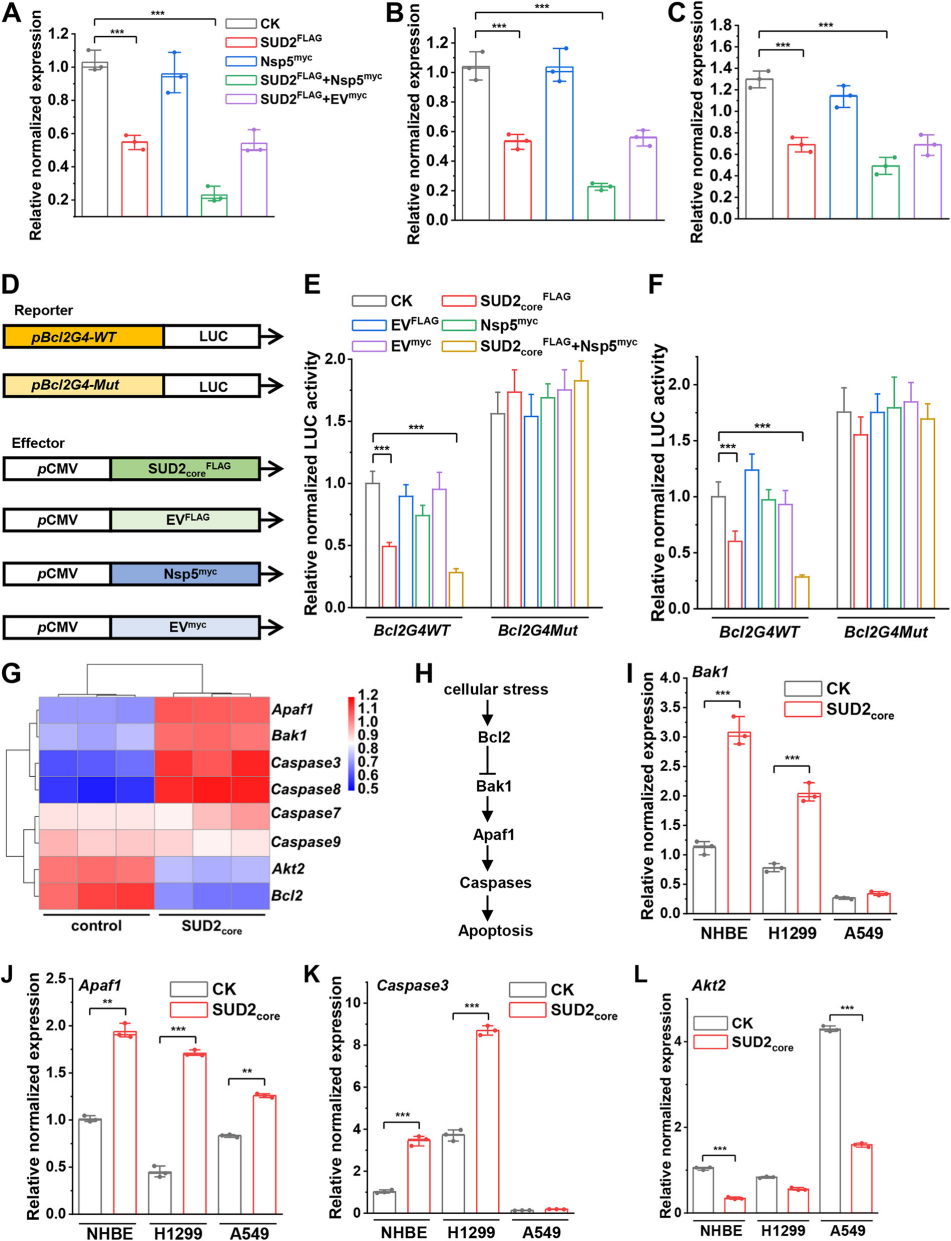
SUD2_core_ and Nsp5 inhibit the expression of BclII in a G4-dependent manner. (A to C) Relative expression of BclII by transfection with SUD2_core_ and Nsp5 in NHBE, H1299, and A549 cells. GAPDH was used as an internal control. (D) Schematic presentation of the reporter and effector constructs. (E and F) The effects of *G4WT* and *G4Mut* in the BclII promoter region on LUC expression in NHBE and H1299 cells. The *Renilla* luminescence reporter activity is expressed relative to the control. EV, empty vector. (G) Heatmap of apoptosis-related gene expression in SUD2_core_-FLAG-transfected NHBE cells 48 h after transfection. (H) BclII-mediated apoptosis-related gene networks. (I to L) Relative expression levels of apoptosis-related genes by transfection with SUD2_core_ in NHBE, H1299, and A549 cells. Data represent the means ± SEM of triplicate biological results. Statistical significance was calculated by unpaired Student's *t* test. **, *P* < 0.01; ***, *P* < 0.001.

As *Bcl2G4* downregulated BclII expression and Nsp5 enhanced the intrinsic binding affinity between SUD2_core_ and *Bcl2G4*, qPCR was performed to evaluate whether BclII expression was altered in the presence of SUD2_core_ versus SUD2_core_-Nsp5 in different respiratory epithelial cells. SUD2_core_ alone reduced the expression of BclII to 53%, 52%, and 56% in NHBE, H1299, and A549 cells, respectively, compared with non-SUD2_core_-transfected cells (Fig. 4A to C), confirming the negative regulatory role of SUD2_core_ on BclII expression. Next, the influence of the SUD2_core_-Nsp5 complex on the expression of BclII was examined. While Nsp5 had a minimal effect on BclII expression, cotransfection of SUD2_core_ and Nsp5 strongly reduced the BclII mRNA level to 21%, 22%, and 38% in NHBE, H1299, and A549 cells, respectively, nearly doubling the effects of SUD2_core_ alone (Fig. 4A to C). Consistent with the low binding affinity of SUD2_core_ for *VEGFR2G4* and *KRASG4*, no significant differences were observed for *VEGFR2* and *KRAS* expression in any of the three cell lines (data not shown). Thus, we showed that SUD2_core_ selectively reduced the expression of BclII in respiratory epithelial cells, and more importantly, SUD2_core_-Nsp5 showed enhanced downregulation of BclII expression compared to SUD2_core_ alone.

To confirm that this regulatory effect is G4 dependent, a dual-luciferase reporter gene assay was performed (Fig. 4D). The results clearly showed that the relative LUC activity of the BclII*-WT* promoter (which formed the G4 structure in NHBE and H1299 cells) was significantly decreased in the presence of SUD2_core_, but not Nsp5 alone, and reached the lowest activity in the presence of both SUD2_core_ and Nsp5, compared with nontreated cells (Fig. 4E and F). In addition, this suppressive effect was lost in the BclII*-Mut* promoter, which could not form G4 structure, confirming that the downregulation of target gene expression by SUD2_core_ and the SUD2_core_-Nsp5 complex is dependent on G4 structure formation.

Cohort studies indicated that decreased BclII expression resulted in the increased death of respiratory cells associated with severe COVID-19 in patients, due to the antiapoptotic role of BclII (33, 35). Since SUD2_core_ has been found to negatively regulate the expression of BclII, to further investigate if this interferes with the expression of other BclII-mediated apoptosis-related genes (Fig. 4H), we performed RNA sequencing (RNA-seq) in NHBE cells in the presence and absence of SUD2_core_ (Fig. 4G). In addition to inhibiting the expression of BclII, SUD2_core_ negatively regulated the BclII-mediated antiapoptotic cascade. Specifically, expression of proapoptotic elements, such as BclII *antagonist/killer 1* (*Bak1*), *apoptotic peptidase activating factor 1* (*Apaf1*), and *Caspase 3/8* were increased, but antiapoptotic *AKT serine/threonine kinase 2* (*Akt2*) was decreased (48, 54, 55). These differential expressions were further confirmed by qPCR (Fig. 4I to L).

### SUD2_core_ and Nsp5 cooperatively promote apoptosis in epithelial cells.

**FIG 5:**
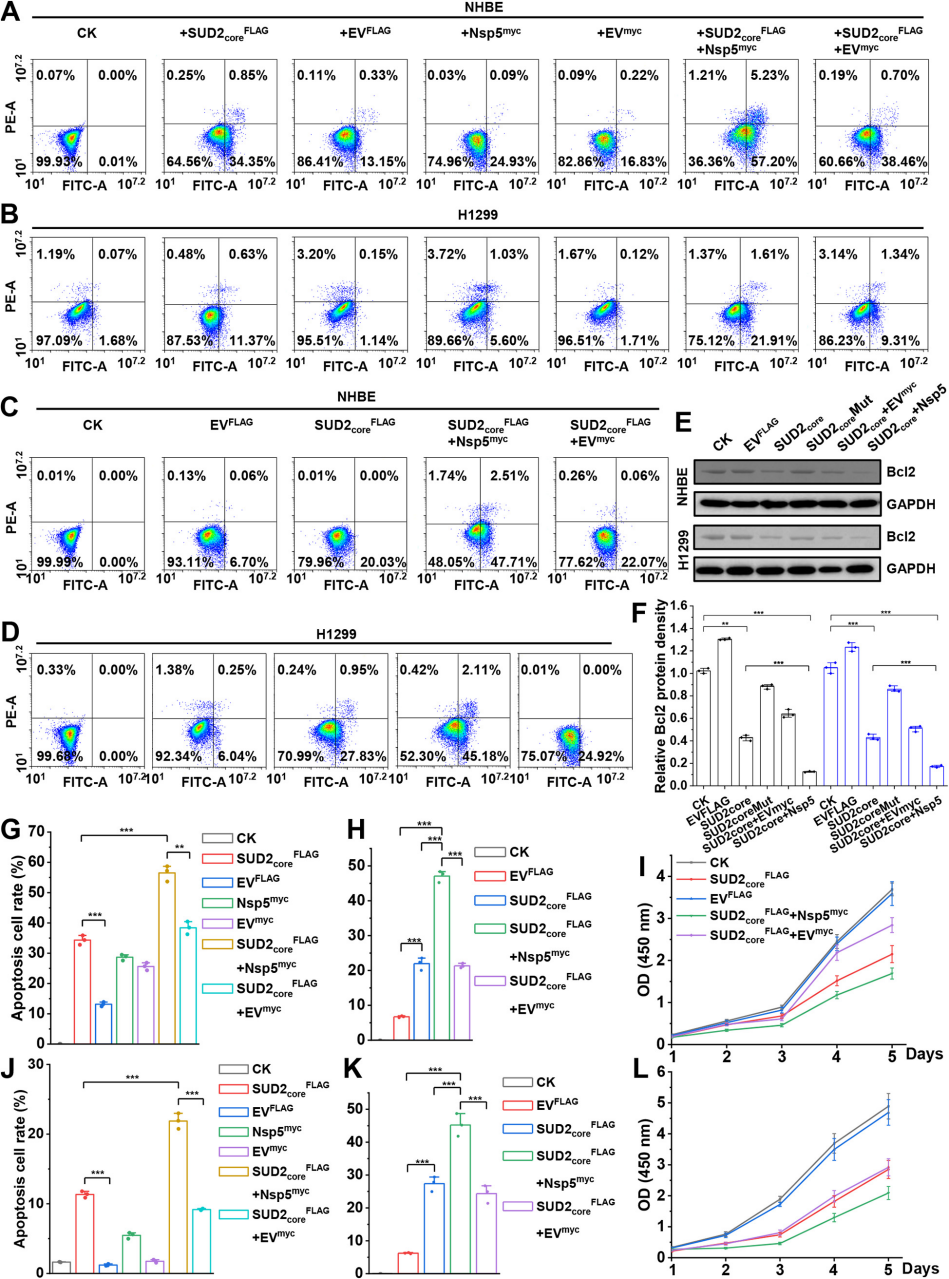
SUD2_core_ and Nsp5 cooperatively promote apoptosis of respiratory epithelial cells. (A and B) Flow cytometry analysis of apoptosis induced by SUD2_core_ and Nsp5 in NBHE and H1299 cells. Fluorescein isothiocyanate (FITC) signal detector and PI staining by the phycoerythrin emission signal detector were used. The Q3-4 displays the apoptosis cells. (C and D) Flow cytometry analysis of apoptosis induced by SUD2_core_ and Nsp5 in SUD2_core_-overexpressing NBHE and H1299 cells. (E) Different protein levels of BclII in NHBE and H1299 cells by introducing different effectors. (F) Relative protein abundance of BclII (corresponding to results shown in panel E). Black and blue columns indicate protein levels in NHBE and H1299 cells, respectively. Data represent the means ± SEM of triplicate biological results. Statistical significance was calculated by unpaired Student's *t* test. **, *P* < 0.01; ***, *P* < 0.001. (G, H, J, and K) Quantitative measurement of apoptosis rates in different cells corresponding to data in panels A, C, B and D, respectively. (I and L) The measurement of cell proliferation of NHBE (I) and H1299 (L) cells transfected with different effectors. The OD_450_ of cells with substrate were determined at the indicated times. Data represent the means ± SEM of triplicate biological results. Statistical significance was calculated by unpaired Student's *t* test. **, *P* < 0.01; ***, *P* < 0.001.

After confirming the inhibitory effect of SUD2_core_ on BclII expression and that the SUD2_core_-Nsp5 complex enhanced this effect, we anticipated an overall proapoptotic biological consequence. To confirm this, the apoptosis rate of respiratory cells was measured by flow cytometry (FCM) after Annexin and propidium iodide (PI) staining. In transient expression systems, the cells underwent minimal apoptosis in the absence of SUD2_core_ and Nsp5. The apoptosis rates were increased up to 2.6-fold and 9.9-fold in the presence of SUD2_core_^FLAG^ in NHBE and H1299 cells, respectively, compared with that with FLAG empty vector (EV^FLAG^) (Fig. 5A, B, G, and J), whereas Nsp5^myc^ alone had little effect on apoptosis rate, confirming the determinant role of SUD2_core_ and the auxiliary role of Nsp5 (Fig. 5A, B, G, and J). In accordance with their enhanced inhibitory effect on BclII expression, the apoptosis rate of NHBE cells in the presence of both SUD2_core_^FLAG^ and Nsp5^myc^ reached 56.5%, 1.5-fold higher than that of cells transfected with SUD2_core_ alone.

Similarly, in the SUD2_core_^FLAG^ stable expression cell lines generated by lentiviral processing, higher apoptosis rates (20.03% and 27.83%) were also observed in NHBE and H1299 cells, compared with that with lentiviral EV^FLAG^ (6.7% and 6.04%) (Fig. 5C, D, H, and K). Moreover, the transient expression of Nsp5^myc^ in the stable SUD2_core_^FLAG^-expressing cells robustly elevated the apoptosis rate, about 2.38-fold and 1.62-fold higher than that of non-Nsp5-treated NHBE and H1299 cells (Fig. 5C, D, H, and K). Immunoblotting confirmed that SUD2_core_ decreased BclII protein levels to about 41% of that with mock control (Fig. 5E and F). After cotransfection of SUD2_core_ and Nsp5, the BclII protein levels decreased dramatically to only 12% and 17% compared with the control NHBE and H1299 cells (Fig. 5E and F). Consistently, the cells transfected with SUD2_core_ and Nsp5 exhibited the lowest proliferation rate compared with both mock control and individually transfected cells (Fig. 5I and L). These results demonstrate that in line with negative regulation on BclII, SUD2_core_-Nsp5 can significantly promote apoptosis in respiratory epithelial cells.

### Structural features of the SUD2_core_-*Bcl2G4* versus SUD2_core_-Nsp5-*Bcl2G4* complex.

**FIG 6:**
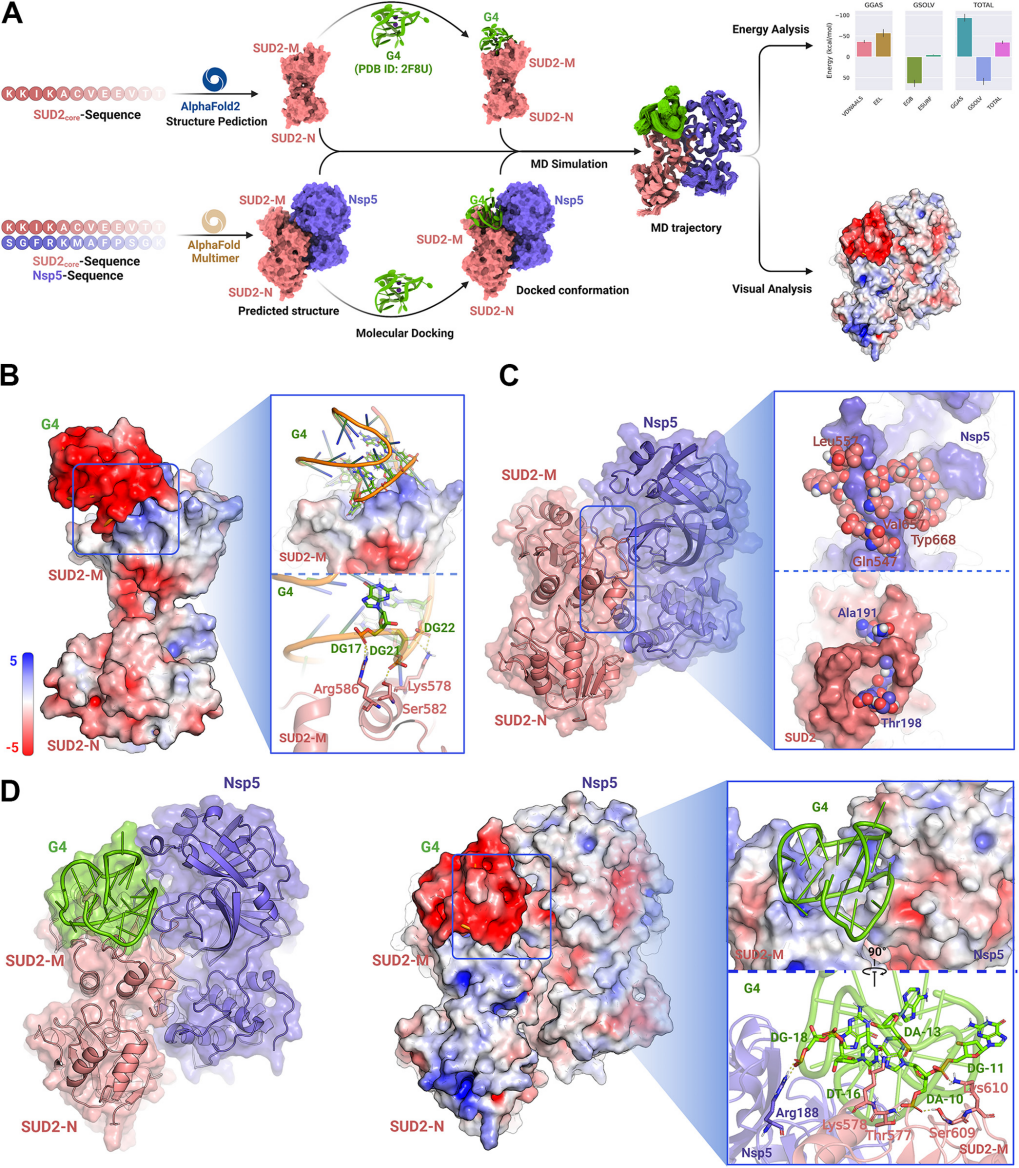
Structural information for SUD2_core_, SUD2_core_-*Bcl2G4*, SUD2_core_-Nsp5, and SUD2_core_-Nsp5-*Bcl2G4*. (A) The flow chart of the protein structural modeling in this study. (B) Representative structure of SUD2_core_-*Bcl2G4* complex modeled with AlphaFold2 algorithm followed by molecular docking. The space-filling electrostatic surface charge distribution showed a positively charged groove on SUD2_core_ bound to negatively charged *Bcl2G4*. The specific interacting residues are displayed in the enlarged inset. (C) The overall structure of the SUD2_core_-Nsp5 complex. The binding surfaces are indicated by red (for SUD2_core_) and blue (for Nsp5) spheres, respectively. (D) The overall structure of the SUD2_core_-Nsp5-*Bcl2G4* tertiary complex.

Since atomic interacting features lay the foundation of the structure-property-function relationship axis of the biological molecules and their complexes, which can also provide crucial information for developing potential intervening measures, after confirmation of the interaction and biological function of SUD2_core_-Nsp5 with *Bcl2G4*, we further investigated the precise interacting surface of the complex and the conformation changes from monomer to ternary complex. Despite many tries, both the monomeric SUD2_core_ and corresponding complexes were refractory to forming suitable crystals for X-ray diffraction analysis. Thus, the final structural information was separately obtained through AlphaFold modeling and use of ZDOCK, followed by molecular dynamics (MD) optimization (Fig. 6A).

The overall structure of the SUD2_core_ monomer was quite similar to SUD1_core_ (PDB code 2W2G) (Fig. S5A), giving strong structural support for their common G4 DNA binding capacity. As a whole, SUD2_core_ is more conformationally unstable than are the individual SUD2-N or SUD2-M subdomains, due to the highly flexible linker between N and M domains, indicated by calculation of the root mean square deviation (RMSD) (Fig. S5B).

10.1128/mbio.03359-22.5 FIG S5 The structural comparison of SUD1_core_ and SUD2_core_ and molecular dynamics simulations of the SUD2_core_ domain. (A) The structural comparison between SUD1_core_ and SUD2_core_. (B) RMSD analysis of SUD2-N, SUD2-M, and SUD2_core_ during 100-ns MD simulations. Red, SUD2-N; green, SUD2-M; blue, SUD2_core_. The RMSD of SUD2-N and M showed higher stability than SUD2_core_. (C) Free energy calculation of SUD2_core_. (Left) Gibbs free energy landscape of SUD2_core_ domain from MD simulations. The axes are the Cα-RMSD and Rg of SUD2_core_. (Right) Representative structures of the expanded and folded states, which displayed 153° and 94° angles between the N and M domains of SUD2. The two conformations could exchange into each other. Download FIG S5, TIF file, 2.2 MB. Copyright © 2023 Li et al. 2023 Li et al. https://creativecommons.org/licenses/by/4.0/ This content is distributed under the terms of the Creative Commons Attribution 4.0 International license.

In the case of the binary SUD2_core_-*Bcl2G4* complex, a positively charged groove at the tip of the M domain of SUD2 was geometrically and electronically optimal for surface interaction with the negatively charged surface of *Bcl2G4* (PDB code 2F8U), in which K578, S582, and R586 of SUD2 would form hydrogen bonds with the phosphodiester backbone of dG22 (2.89 Å), dG21 (2.72 Å), and dG17 (2.49 Å) of *Bcl2G4* DNA, respectively (Fig. 6B). These three amino acids also displayed low free energy in the presence of *Bcl2G4* (Fig. S6A to D). Based on this information, we expressed and purified the mutation of SUD2_core_-Mut (K578A, S582A, and R586A) and found that the mutant protein domain did significantly reduce the binding affinity with *Bcl2G4* DNA (Fig. 3E and F).

10.1128/mbio.03359-22.6 FIG S6 Molecular dynamics simulation of the SUD2_core_-*Bcl2G4* complex. (A) RMSD analysis of SUD2-N, SUD2-M, and SUD2_core_-*Bcl2G4* during 100-ns MD simulations. Orange, *Bcl2G4*; red, SUD2-N; green, SUD2-M; blue, SUD2_core_-*Bcl2G4*. Even though the conformational change existed between the N and the M domain, *Bcl2G4* could still stably bind in the M domain. (B) The fluctuations and the accumulated mean values of entropies for SUD2_core_-*Bcl2G4*. (C) The contributions of GGAS and GSOLV decomposed by the binding free energy between SUD2_core_ and *Bcl2G4*. GGAS is the interaction energy and is obtained after summing the internal components and the nonbonded components. For GSOLV, the polar and nonpolar contributions are EGB and ESURF, respectively, for GB calculations. (D) Decomposition of free energy on a per-residue basis into contributions from the nonpolar interaction energy and polar solvation energy for residues in the SUD2_core_-*Bcl2G4* complex. Download FIG S6, TIF file, 2.2 MB. Copyright © 2023 Li et al. 2023 Li et al. https://creativecommons.org/licenses/by/4.0/ This content is distributed under the terms of the Creative Commons Attribution 4.0 International license.

A well-fitted heterodimeric structure of the SUD2_core_-Nsp5 complex was obtained by using the AlphaFold2 multimer algorithm, in which two complementary surfaces crossing the N and M domains of SUD2 mediate the crucial interactions with Nsp5: (i) a groove in the SUD2-N domain bound to a wedge of Nsp5 containing the amino acid residues from A191 to T198 of Nsp5 was identified; (ii) another binding pocket was determined to occur between a wedge of SUD2-M containing amino acid residues Q547, L557, V657, and Y668 and a groove of Nsp5 (Fig. 6C and Fig. S7B to D). Therefore, in this complexation manner, Nsp5 could stabilize the lowest-energy conformation of SUD2_core_ (closed status exhibiting 94° between N and M domain) and prevent the conformational exchange (Fig. S5C). Consistently, in MD analysis, the SUD2_core_-Nsp5 complex was more stable than the SUD2_core_ monomer (Fig. S5B and S7A). Together, the structural modeling results mesh very well with the affinity experiment with truncated proteins described above (Fig. 1D).

10.1128/mbio.03359-22.7 FIG S7 Molecular dynamics simulation of SUD2_core_-Nsp5 complex. (A) RMSD analysis of SUD2_core_, Nsp5, and SUD2_core_-Nsp5 during 100-ns MD simulations. Red, Nsp5; green, SUD2_core_; blue, SUD2_core_-Nsp5. (B) The fluctuations and the accumulated mean values of entropies for SUD2_core_-Nsp5 complex. (C) The contributions of GGAS and GSOLV decomposed by the binding free energy between SUD2_core_ and Nsp5. (D) Decomposition of free energy on a per-residue basis into contributions from the nonpolar interaction energy and polar solvation energy for residues in the SUD2_core_-Nsp5 complex. Download FIG S7, TIF file, 2.2 MB. Copyright © 2023 Li et al. 2023 Li et al. https://creativecommons.org/licenses/by/4.0/ This content is distributed under the terms of the Creative Commons Attribution 4.0 International license.

For the tertiary SUD2_core_-Nsp5-*Bcl2G4* complex, *Bcl2G4* was calculated to interact well in the original pocket, albeit with altered interaction numbers and positions of SUD2_core_: T577, K578, S609, and K610 formed hydrogen bonds with the phosphodiester backbone of dA13, dT16, dA10, and dG11 of *Bcl2G4* DNA, respectively (Fig. 6D). In addition to these SUD2_core_-*Bcl2G4* interactions, there was an added interaction between the dG18 arm of *Bcl2G4* and Arg188 of Nsp5 (Fig. 6D). The space-filling electrostatic surface charge distribution also showed that a positively charged groove on the SUD2_core_-Nsp5 complex when bound to a negatively charged *Bcl2G4* resulted in a more stable insertion of G4 into SUD2_core_ (Fig. 6D). The free energy analysis indicated a much lower averaged total free energy of SUD2_core_-Nsp5-*Bcl2G4* (~90 kcal/mol) compared with SUD2_core_-*Bcl2G4* (~40 kcal/mol) (Fig. S6B and S8A to D), which could explain the experimental observation that Nsp5 enhanced the binding of SUD2_core_ with *Bcl2G4* (Fig. 3F and G).

10.1128/mbio.03359-22.8 FIG S8 Molecular dynamics simulation of SUD2_core_-Nsp5-*Bcl2G4* complex. (A) RMSD analysis of SUD2_core_-Nsp5-*Bcl2G4* during 100-ns MD simulations. Orange, *Bcl2G4*; green, SUD2_core_; red, Nsp5; blue, SUD2_core_-Nsp5-*Bcl2G4*. (B) The fluctuations and the accumulated mean values of entropies for SUD2_core_-Nsp5-*Bcl2G4* complex. (C) The contributions of GGAS and GSOLV decomposed by the binding free energy between SUD2_core_-*Bcl2G4* and Nsp5. (D) Decomposition of free energy on a per-residue basis into contributions from the nonpolar interaction energy and polar solvation energy for residues in the SUD2_core_-Nsp5-*Bcl2G4* complex. Download FIG S8, TIF file, 2.2 MB. Copyright © 2023 Li et al. 2023 Li et al. https://creativecommons.org/licenses/by/4.0/ This content is distributed under the terms of the Creative Commons Attribution 4.0 International license.

Therefore, the geometric complementarity and intermolecular and electrostatic interactions together account for the enhanced stability of the tertiary complex of SUD2_core_-Nsp5-*Bcl2G4*, compared to those for the binary complex of SUD2_core_-*Bcl2G4*.

## DISCUSSION

The discovery of the *SUD1* sequence traces back to the SARS epidemic in 2003 (15). Later, the SUD1 domain of SARS-CoV-2 Nsp3 was reported to bind with oligo G nucleotides, especially G4 structural DNA, which may result in the severe pathogenicity of SARS-CoV (17, 18, 44). In the COVID-19 pandemic, the similar *SUD2* sequence was again identified in the genome of SARS-CoV-2 (30). Recently, Lavigne et al. biophysically confirmed that the monomeric SUD2_core_ of SARS-CoV-2, just like SUD1, can also bind with some G4s structures *in vitro* (20). However, two aspects surrounding SUD2_core_ need further investigations. First, whether its interaction with DNA G4 structure exists in host cells remains to be confirmed, and the biological consequences in the host cells of this kind of interaction were not fully examined. Second, in addition to individual components, protein-protein interactions (PPI) mediated by embedded domains are of fundamental importance for understanding the disease mechanism and development of efficient intervention options (56–58). In the case of COVID-19, the elucidation of the interactions between viral and host proteins has led to the identification of some aspects of the infectious mechanism and potential effective therapies, including antibody and small-molecule agents (59). Apart from viral-host PPI, the interplay between the viral proteins themselves also play crucial roles in the life cycle of SARS-CoV-2. For instance, Nsp7 and Nsp8 help Nsp12 to make new copies of the RNA genome for assembling new viruses (60). However, how SUD2_core_ cooperates with other Nsp’s to exert its pathological effect was unknown.

Surrounding these two aspects, in this study new mechanistic and structural information regarding such interactions *in vitro* and in cells was provided, not just for SUD2_core_ monomer but also for its viral protein complex. We used a Y2H system to screen SUD2_core_ binding partners in Nsp’s of SARS-CoV-2; Nsp5 was identified as the sole partner of SUD2_core_, and their binding affinity (*K_D_* = 37.4 nM) was quantitatively measured *in vitro*. The intracellular interaction between SUD2_core_ and Nsp5 was confirmed and detected in the nucleus of respiratory epithelial cells, suggesting that SUD2_core_ and Nsp5 indeed formed a complex in host cells. Moreover, we validated that SUD2_core_ tightly binds with *Bcl2G4* DNA and Nsp5 enhances this interaction. Consequently, this resulted in reduced BclII expression and an enhanced apoptosis rate of respiratory epithelial cells. The structural features of this complex, including detailed residue interactions and the more stable conformation of this protein-G4 DNA tertiary complex, have been rationalized through theoretical modeling and MD analysis.

Regarding the binding target, sequencing data and bioinformatic studies have revealed the prevalence of many G4 motifs at gene regulatory regions in the human genome (45, 46). Despite the guanine tetrad serving as the common inner core unit of G4 quadruplexes, distinct topologies and local loop structures have been found for many different G4 sequences. This G4 structural diversity controls their interactions with other components and the resulting gene regulation effects. Though a certain extent selective binding of SUD2_core_ with G4 sequences has been observed (i.e., *Bcl2G4* versus *VEGFR2G4* and *KRASG4*), considering the huge number of G4 sequences existing at different loci of the human genome (which cannot be exhaustively investigated at one time), the interaction of SUD2_core_ with G4s in other gene regulatory regions cannot be excluded. For instance, as mentioned above, SUD2_core_ can also bind to *MYCG4* (20). In line with this deduction, from GO and KEGG enrichment analysis of comparative RNA-seq data, SUD2_core_ also regulates the expression of genes involved in other biological processes and pathways, such as DNA binding, ISG15-protein conjugation, herpes simplex virus 1 infection, and interleukin-17 signaling pathway (Fig. S9). Regarding the complexity of transcription regulation, among these alterations induced by SUD2_core_, further investigation is needed to determine which ones are the direct consequence of SUD2_core_-*Bcl2G4* interaction.

10.1128/mbio.03359-22.9 FIG S9 GO and KEGG scatter diagram and heatmap of differentially expressed genes in SUD2_core_-overexpressing NHBE cells compared to control cells. (A and B) GO and KEGG enrichment analysis for differentially expressed genes. The horizontal axis represents the enrichment degree (rich factor; representing the number of differential genes belonging to a certain GO or KEGG term dividided by the total number of genes belonging to this GO or KEGG term), and the vertical axis represents the enrichment of GO or KEGG term. Dot size indicates the number of differential genes enriched in a certain GO or KEGG term. Dot colors indicate different *P* values. (C) For nonbiological duplications, the log_10_(FPKM + 1) was used to display, and for biological duplications, the z-value method was used to display gene expression. The color changes from blue through white to red indicate the expression level changes from low to high. Download FIG S9, TIF file, 2.8 MB. Copyright © 2023 Li et al. 2023 Li et al. https://creativecommons.org/licenses/by/4.0/ This content is distributed under the terms of the Creative Commons Attribution 4.0 International license.

The molecular modeling and docking features of the SUD2_core_-*Bcl2G4* complex predicted in this study are different from those in a previous report (20). Here, a positively charged groove at the top of the M domain of SUD2 interacted with a negatively charged surface of *Bcl2G4*, in which hydrogen bonds formed between K578, S582, and R586 of SUD2 and the phosphodiester backbone of *Bcl2G4* were detected. In comparison, in a previous report, *Bcl2G4* docked at the SUD-N–SUD-M interface and comprised residues K592 and E595 (20). Two reasons might account for this discrepancy. First, in Lavigne’s study a dimeric SUD2-NM was employed for the docking calculation, and we chose the monomeric unit, considering our screening, biophysical, and biochemical experiments. Second, different algorithms of computational performance were employed. In our study, the conformation search algorithm was performed by the fast Fourier transform in the ZDOCK program, which mainly aims at the docking of protein-protein or protein-nucleic acid complexes (61). In the previous study, the conformation search algorithm used was a stochastic global optimization with the Autodock-vina program, which is more commonly used for small-molecule–protein complexes (62). To verify our calculated result, the substitution of K578A, S582A, and R586A of SUD2 was generated for the BLI assay, which indicated that these three amino acids did play important roles in binding with *Bcl2G4*. Surely, the possibility of multiple G4 binding sites of SUD2_core_ may exist, and the roles of the K592 and E595 residues predicted in the previous study for G4 binding deserve further investigation.

Considering SUD2 as an individual functional domain, in addition to participating in G4 DNA binding, it was also characterized to enhance viral RNA translation (20, 30). As a part of the largest nonstructural protein, the tracing back to and investigating the biological function of full-length Nsp3 is an important matter from a holistic aspect (30). Unfortunately, due to technical limitations, we could not obtain the full length of Nsp3 or transfect it into host cells (63, 64). Note that other previous studies also failed to express full-length Nsp3, and thus only the Nsp3-N was investigated. In a previous report, it was found that unlike discrete, punctate nuclear localizations found for Nsp1, Nsp5, Nsp9, and Nsp13, Nsp3-N was both cytoplasmic and rather diffusely found across the nucleus, indicating that Nsp3 may be involved in DNA transcription and genomic regulation (42). Our subcellular localization result for SUD2_core_ was consistent with Nsp3-N. In addition, we have found a nuclear localization sequence (KKAGGTTEMLAKALRKV) in the SUD2 domain (and hence also in Nsp3) (65); therefore, it can be inferred that the full Nsp3 may also have the capability to enter the nucleus. This deduction needs further experimental validation, and how the SUD2_core_ domain will perform in the milieu of full-length Nsp3 protein awaits further investigation.

In addition to the SUD2 domain, Nsp3 contains multiple modular protein domains, and these portions have also been investigated separately and display different roles during viral infection in host cells. For instance, the Mac1 domain removes ADP ribosylation posttranslational modifications (66), and the PL_pro_ domain antagonizes MDA5-mediated type I interferon signaling to suppress the initial immune response (67).

For the other complex components, apart from being the partner of SUD2_core_, Nsp5 has also been previously identified as a main protease in the SARS-CoV-2 genome and is essential for cleaving the replicase polypeptides (pp1a and pp1ab) and the replication and transcription of SARS-CoV-2. Therefore, further research is needed to address how the interaction between SUD2 and Nsp5 influences the function of Nsp5 in this role. Despite these limitations, our current study provides new information that the SUD2_core_-Nsp5-*Bcl2G4* interaction leads to increased apoptosis of respiratory epithelial cells, which has been identified as a hallmark of severe COVID-19 disease. Based on these results, new therapies targeting SUD2, Nsp5, destabilizing G4, or their mutual interfaces can be envisioned.

## MATERIALS AND METHODS

### Plasmid construction.

For the Y2H assay, the coding sequences (CDS) of all Nsps of SARS-CoV-2, except Nsp3, were synthesized by Tsingke commercial company, then cloned into the pGADT7 vector (Clontech catalog number 630442) by using NdeI and BamHI. The CDS of SUD2_core_ was cloned into pGBKT7 vector (Clontech catalog number 630443) by using EcoRI and SalI. For co-IP, RNA-seq, ChIP-qPCR, and apoptosis assays, SUD2_core_ was generated into a pcDNA3.1-3FLAG vector (Thermo Fisher Scientific catalog number V79020) and packed with lentiviruses by PackGene Co. The pcDNA3.1-SUD2_core_-GFP plasmid was constructed by using EcoRI and BamHI, and pcDNA3.1-Nsp5-GFP was constructed by using BamHI and EcoRI for subcellular localization. For BiFC experiments, SUD2_core_ was fused to pBiFC-VN173 vector (Addgene catalog number 22010) containing the YFP^N^ fragment, and Nsp5 was fused to pBiFC-VC155 vector (Addgene catalog number 22011), respectively. For the co-IP assay, SUD2_core_ and Nsp5 were fused into pcDNA3.1-SUD2_core_-3FLAG and pcDNA3.1-Nsp5-GFP, respectively. For expression of recombinant SUD2 and Nsp5 in E. coli, CDS of SUD2_core_ was introduced into pET-28a(+) expression vector (Novagen catalog number 69866-3) by using BamHI and SalI and fused with His tag. Nsp5 was introduced into pGEX6P-1 expression vector (GE catalog number 28-9546-48) by using BamHI and EcoRI, which fused with GST tag. For the firefly luciferase assay, the DNA region located 2,000 bp upstream of the translational start site of the BclII gene, including the G4 sequences (*pBcl2*-*WT* or *pBcl2*-*Mut*), was introduced into the pNLCoI1 (luc2-P2A-NlucP/Hygro) vector (Promega catalog number N1461) to analyze the expression of BclII in human respiratory cells.

### Cell culture and transfection.

Normal human bronchial epithelial cells (NHBE) were purchased from Lonza, and human lung epithelial cells (A549 and H1299) were purchased from Shanghai Cell Bank, Chinese Academy of Sciences. NHBE cells were cultured in Dulbecco’s modified Eagle’s medium, and A549 and H1299 cells were cultured in RPMI 1640, containing 10% fetal bovine serum, 100 mg of streptomycin/mL, and 100 U of penicillin/mL. The cells were maintained in a chamber at 37°C with 5% CO_2_.

For cell transfections, the cells were cultured after 10 generations and then plated in 35-mm confocal dishes at about 200,000 cells per dish. One microgram amounts of plasmids of SUD2_core_, Nsp5, or SUD2_core_ plus Nsp5 were added to the cells with Lipofectamine 3000 reagent according to the manufacturer’s instructions.

### Yeast two-hybrid assay.

Y2H was performed following the instructions for the Yeastmaker yeast transformation system 2 (Clontech manual PT1172-1). Generally, AD-Nsps and BD-SUD2_core_ were cotransformed into the yeast strain Y2HGold using the polyethylene glycol-lithium acetate method (manual PT1172-1). All of the clones grew well on SD medium minus leucine and tryptophan (-LW). The positive clones were identified by the ability to grow on SD medium minus leucine, tryptophan, and histidine (-LWH). The photographs were taken after 3 days. The primers that were used in this assay are listed in Table S1.

For β-galactosidase activity assays, Y187 cells were grown to mid-log phase (optical density at 600 nm [OD_600_] of 0.5). The cell pellets were resuspended in Z buffer (60 mM Na_2_HPO_4_·7H_2_O, 40 mM NaH_2_PO_4_·H_2_O, 10 mM KCl). In order to permeabilize the cells, suspensions were subjected to 3 freeze and thaw cycles before adding *o*-nitrophenyl-β-d-galactopyranoside (ONPG). After incubation, the OD_420_ of the samples was measured in a spectrophotometer, and β-galactosidase units were calculated according to the following equation: (1,000 × OD_420_)/(*t* × *V* × OD_600_), where *t* is the time (in minutes) to the appearance of yellow color after adding the ONPG, and *V* is the volume (in milliliters) of cell culture used.

### Co-IP assay.

The pcDNA3.1-Nsp5-GFP, pcDNA3.1-GFP empty vector, and pcDNA3.1-SUD2_core_-3FLAG plasmids were transfected into human epithelial cells for the co-IP assay. First, Dynabeads-protein G was incubated with anti-FLAG antibody or anti-GFP antibody at 4°C for 1 h. After that, the total proteins of different cells were extracted and then put on ice with IP buffer (20 mM HEPES [pH 7.5], 200 mM NaCl, 10 mM MgCl_2_, 0.1% Triton X-100, 1 mM EDTA, 10% glycerol, 10 mM protease inhibitor cocktail, and 2 mM phenylmethylsulfonyl fluoride). The supernatants were incubated with antibody-protein G complexes at 4°C for 4 h. The beads were washed 5 times with IP buffer. Then, the samples were loaded on SDS-PAGE and detected with anti-FLAG antibody or anti-GFP antibody.

### Subcellular localization and BiFC assay.

For subcellular localization, the SUD2_core_-GFP or Nsp5-GFP plasmids were transfected into NHBE cells, and the GFP signal was investigated after 24 h. Hoechst staining indicated the nuclei of epithelial cells. Photos were captured by a confocal fluorescence microscope (Stellaris; Leica, Germany). For the BiFC assay, enhanced YFP signaling (VENUS) was captured using the same instrument.

### Protein expression and purification.

The sequenced plasmids of pET28a (+)-SUD2_core_, SUD2 _core_-Mut, and pGEX6P-1-Nsp5 were transformed into the E. coli expression strain BL21(DE3). For the protein expression procedure, 0.5 mM isopropyl-β-d-thiogalactoside was added to fresh E. coli cells and cultured overnight at 16°C. After sonification and centrifugation, cell pellets were collected in binding buffer (50 mM Tris-HCl [pH 8.0], 300 mM NaCl, and 5 mM imidazole), His-SUD2_core_ was eluted by 200 mM imidazole in a His-labeled nickel column, and Nsp5-GST proteins were eluted by 10 mM reduced glutathione in a GST-agarose resin. The eluted proteins were then added into an Amicon Ultra-15 ultrafiltration tube (Merck catalog number UFC901096) for concentration and desalting. Separation and purification were performed by HITRAP Q-affinity chromatography and Superdex 200 Increase 3.2/300 gel filtration chromatography (Cytiva). SDS-PAGE was used to detect the above proteins. The charge properties and polymerization degree of the proteins were uniform and the purity reached 95% for further study.

### CD spectroscopy.

The single-strand *Bcl2G4*, *VEGFR2G4*, and *KRASG4* DNAs were synthesized by Sango Biotechnology in high-performance liquid chromatography level. The oligonucleotides were folded into G4s by the following procedure: 95°C heating in 50 mM Tris-HCl (pH 8.0), 100 mM KCl for 5 min, then slowly cooled to room temperature. The linear DNA was annealed without K^+^ buffer. The oligonucleotides were then diluted to 1 μM, and 1.5 mL of the reaction solution was mixed in a 1-cm optical-path-length quartz colorimetric dish and then measured directly. The measurements recorded CD values between 220 nm and 300 nm at room temperature. Each CD spectrum curve represented the average of 10 measurements and was eventually smoothed by a Savitsky-Golay filter, using the CD instrument (Chirascan Plus, Applied Photophysics, UK). If an annealed oligonucleotide chain showed a trough at 240 nm and a peak at 260 nm, it could form a positively parallel G4 structure.

### Biolayer interferometry assay.

Binding kinetic analyses of SUD2_core_-Nsp5 and SUD2_core_-*Bcl2G4* were performed by BLI at ForteBio Octet (Sartorius, Germany). For detection of the SUD2_core_-Nsp5 interaction, the purified His-tagged SUD2_core_ was immobilized onto anti-His antibody biosensors (sensors were hydrated for 10 min before use) in a BLI buffer (50 mM Tris-HCl [pH 8.0], 100 mM KCl, 0.05% Tween 20, and 5% [vol/vol] glycerol). It was then incubated with Nsp5 protein as follows: baseline for 60 s, loading for 90 s, association for 180 s, and dissociation for 300 s. For detection of the SUD2_core_-*Bcl2G4* interaction, the G4 structure of biotin-labeled *Bcl2G4* was immobilized onto the SA biosensors in the BLI buffer. Then, it was incubated with SUD2_core_ as follows: baseline for 60 s, loading for 90 s, association for 90 s, and dissociation for 150 s. The reference sensors without His-tagged SUD2_core_ or biotin-labeled G4 DNA served as background controls. Curves were fitted to a 1:1 interaction model, and the *K_D_* value was calculated using Octet data analysis studio software (Sartorius, Germany).

### Luciferase reporter activity assay.

The dual luciferase reporter assay was performed according to the manufacturer’s instructions (Promega TM058). In brief, 80 μL of Dual-Glo reagent was added to each cell culture medium to lyse cells for 10 min, then the firefly luminescence was measured in a luminometer (Victor Nivo Alpha S, PerkinElmer, USA). After that, 80 μL of Dual-Glo Stop & Glo reagent was added to measure the *Renilla* luminescence. Relative response ratios were calculated from the normalized ratios.

### EMSA.

Purified SUD2_core_ (200 ng) was incubated with 10 ng Cy5-labeled G4 probe in EMSA buffer (20 mM Tris-HCl [pH 7.9], 5% [vol/vol] glycerol, 200 mM MgCl_2_, 0.1 mM dithiothreitol [DTT], 4% [wt/vol] bovine serum albumin [BSA], and 0.05% [wt/vol] salmon sperm DNA) for 20 min at 4°C. A 10% nondenaturing polyacrylamide gel was prepared for electrophoresis. The FujiFilm Starion fla-9000 system was used to detect the fluorescent signal after electrophoresis, and the excitation light and emission light were 646 and 664 nm, respectively. The mobility of the probe was calculated.

### G4 pulldown assay.

G4 pulldown assays were performed as described previously (20). In brief, 5′-biotin labeled DNAs (Table S1) were folded into G4s as follows: 95°C heating in 50 mM Tris-HCl (pH 8.0), 100 mM KCl for 5 min, then slow cooling to room temperature. Streptavidin magnetic beads were equilibrated with G4s in binding buffer (50 mM Tris-HCl [pH 8.0], 100 mM KCl, 0.1 mM EDTA, 1 mM DTT, and 0.05% Tween) for 1 h. Then, the purified SUD2_core_ was incubated with the G4-streptavidin magnetic bead complexes for 4 h at 4°C. The beads were washed 5 times before the samples were loaded for SDS-PAGE, and detection was with anti-His antibody (1:5,000; Genscript catalog number A00186).

### Atomic modeling analysis.

For three-dimensional (3D) structure predictions of proteins, the structures of SUD2_core_ and SUD2_core_ in complex with Nsp5 were predicted using the AlphaFold2 and AlphaFold2-multimer programs, respectively. The conformation with the highest score was used for subsequent molecular docking, dynamic simulation, or analysis.

For molecular docking, the structure of *Bcl2*G4 was obtained from the PDB database (PDB ID 2F8U). Subsequently, *Bcl2G4* was docked to the just-predicted SUD2_core_ and SUD2_core_-Nsp5 complexes using the ZDOCK online server (https://zdock.umassmed.edu/) to predict their binding mode.

For molecular dynamics simulation, GROMACS package (version 2021.03) was applied to run conventional MD simulations to investigate the changes in conformation of SUD2 alone and binding modes of SUD2_core_-G4 and SUD2_core_-Nsp5-G4 complexes. The force fields amber14sb and OL15 ff were employed to parameterize protein and G4, respectively. The TIP3P was used for the waters. The protein or protein-G4 complex were solvated in an octahedral water box, and then the charge of the system was neutralized by adding 0.150 M chloride and sodium ions. First, the steepest descent minimization method was used to minimize the energy of the system by 50,000 steps. In the next step, we restricted the position of heavy atoms to run both constant number of atoms, volume and temperature (NVT) equilibration and constant number of atoms, pressure and temperature (NPT) equilibration by 50,000 steps. The system temperature was maintained at 300 K, and the system pressure was maintained at 10^5^ pa. Upon completion of the two equilibration phases, the system was considered well-equilibrated at the desired temperature and pressure. A 100-ns unrestrained simulation was carried out. Every 10 ps, the energy and coordinate system of the trajectory was saved. In the simulation trajectory, ChimeraX and PyMOL were used to map interaction patterns and animate kinetic trajectories. To study the conformational changes of SUD2_core_, we performed PCA processing of molecular dynamics trajectories using the covar, anaeig, and sham commands that come with GROMACS to describe the magnitude of various conformational free energies of macromolecules.

### Free energy calculations and residue decomposition.

The MM-GBSA method has been widely adopted in the estimation of binding free energy in drug research. In our work, the MM-GBSA calculation was performed using the gmx_MMPBSA, a tool of GROMACS for MM-PB (GB) SA calculations. To understand the binding of protein and G4 at the molecular level, we used gmx_MMPBSA to decompose the free energy of binding to the contribution of each residue to the free energy of binding.

### ChIP-qPCR assay.

About 5 × 10^7^ well-grown cells were cross-linked by formaldehyde to a final concentration of 0.75% and were rotated gently at room temperature for 10 min. Glycine (125 mM) was added for 5 min at room temperature, and then cells were rinsed twice with 10 mL cold phosphate-buffered saline (PBS) and added to 5 mL of cold PBS. Cells were thoroughly scraped into a 50-mL tube and then centrifuged for 5 min at 4°C, 1,000 × *g*. The pellets were resuspended in ChIP lysis buffer (50 mM HEPES-KOH [pH 7.5], 140 mM NaCl, 1 mM EDTA [pH 8.0], 1% Triton X-100, 0.1% sodium deoxycholate, 0.1% SDS, and 1× protease inhibitors) and incubated for 10 min on ice.

The samples were then sonicated using a BioruptorPico (Diagenode, Belgium) for 10 min with the following parameters: duty factor, 8%; intensity peak power, 120; cycles per burst, 200; bath temperature, 4°C. The DNAs were sheared to an average fragment size of 200 to 1,000 bp. After sonication, cell debris was pelleted by centrifugation for 10 min, 4°C, 8,000 × *g*. Supernatants of chromatin were incubated with elution buffer (1% SDS and 100 mM NaHCO_3_) plus NaCl and RNase A while shaking at 65°C overnight. Proteinase K (20 mg/mL) was added and incubated while shaking at 60°C for 1 h. DNAs were purified by using a PCR purification kit (QIAamp DNA Micro kit, Qiagen catalog number 56304). A 25-μg amount of purified DNA was diluted 1:10 for each sample (one sample for anti-FLAG antibody and one sample for the beads-only control) with RIPA buffer (50 mM Tris-HCl [pH 8.0], 150 mM NaCl, 2 mM EDTA [pH 8], 1% NP-40, 0.5% sodium deoxycholate, 0.1% SDS, and 1× protease inhibitors). Fifty microliters of chromatin was removed to serve as input sample. Then, anti-FLAG antibody was added to all samples except the beads-only control and rotated at 4°C for 1 h. Equal volumes of washed protein G beads, single-stranded herring sperm DNA, and BSA were added to a final concentration of 75 ng/μL of beads. BSA was also added to a final concentration of 0.1 μg/μL of beads. A 60-μL aliquot of blocked protein A/G beads was added to all samples and immunoprecipitated overnight with rotation at 4°C. Then, the immunoprecipitated samples were centrifuged for 1 min, 2,000 × *g*, and the supernatants were removed.

The pellets were washed with a low-salt wash buffer (0.1% SDS, 1% Triton X-100, 2 mM EDTA, 20 mM Tris-HCl [pH 8.0], 150 mM NaCl), high-salt wash buffer (0.1% SDS, 1% Triton X-100, 2 mM EDTA, 20 mM Tris-HCl [pH 8.0], 500 mM NaCl), and LiCl wash buffer (0.25 M LiCl, 1% NP-40, 1% sodium deoxycholate, 1 mM EDTA, 10 mM Tris-HCl [pH 8.0]). The pellets were collected by centrifugation for 1 min, 2,000 × *g*. The washed DNA was eluted by 120 μL of elution buffer (1% SDS, 100 mM NaHCO_3_), incubated with protein A/G beads, and vortexed for 15 min at 30°C. The supernatants were collected by centrifuging for 1 min at 2,000 × *g*, then NaCl and RNaseA were added while shaking at 65°C overnight. Proteinase K was added to incubate with shaking at 60°C for 1 h. The DNAs were purified by using a PCR purification kit and served as qPCR templates. The abundances of BclII*-G4* promoter regions were detected by qPCR using specific primers.

### RNA-sequencing.

Total RNA was isolated and purified using TRIzol reagent, and the RNA integrity was assessed by using the Bioanalyzer 2100 system (Agilent, CA, USA) with a RNA integrity number (RIN) number of >7.0. Poly(A) RNA was purified from 1 μg of total RNA using Dynabeads Oligo(dT) 25-61005 (Thermo Fisher Scientific) for two rounds of purification. Then, the poly(A) RNA was fragmented into small pieces using the Magnesium RNA fragmentation module (NEB catalog number e6150) under 94°C for 5 to 7 min. Then, the cleaved RNA fragments were reverse-transcribed to create the cDNA by using SuperScript II reverse transcriptase (Thermo Fisher Scientific catalog number 1896649). These were then used to synthesize U-labeled second-stranded DNA with E. coli DNA polymerase I (NEB catalog number m0209), RNase H (NEB catalog number m0297), and dUTP solution (Thermo Fisher Scientific catalog number R0133). Single or dual index adapters were ligated to the fragments. After heat-labile UDG enzyme (NEB catalog number m0280) treatment of the U-labeled second-stranded DNAs, the ligated products were amplified with PCR as follows: initial denaturation at 95°C for 3 min; 8 cycles of denaturation at 98°C for 15 s, annealing at 60°C for 15 sec, and extension at 72°C for 30 sec; and then final extension at 72°C for 5 min. The average insert size for the final cDNA library was 300 ± 50 bp. Next, 2 × 150-bp paired-end sequencing (PE150) was performed on an Illumina Novaseq 6000 system (LC-Bio Technology Co., Ltd., Hangzhou, China) following the vendor's recommended protocol.

After removing the lower-quality bases and undetermined bases, HISAT2 software was used (hisat2-2.0.4) to map reads to the genome. The mapped reads of each sample were assembled using StringTie with default parameters. All transcriptomes from all samples were merged to reconstruct a comprehensive transcriptome using gffcompare software. After the final transcriptome was generated, StringTie and ballgown were used to estimate the expression levels of all transcripts and determine expression levels for mRNAs by calculating the fragments per kilo base per million mapped reads (FPKM). The differentially expressed mRNAs were selected with a fold change of >2 or fold change of <0.5 and a *P* value of <0.05 by R package edgeR or DESeq2, and then GO enrichment and KEGG enrichment were performed to analyze the differentially expressed mRNAs.

### Quantitative real-time PCR analysis of gene expression.

Total RNA was extracted from 10th-generation cells using TRIzol reagent (Thermo Fisher Scientific catalog number 15596026), and reverse transcription was performed using the HiScript II 1st Strand cDNA synthesis kit (Vazyme catalog number R212-01). All of the qPCR experiments for the analysis of differential gene expression were performed using the QuantStudio 3D instrument and the reagent PowerUp SYBR green (Applied Biosystems). Glyceraldehyde-3-phosphate dehydrogenase (GAPDH) was used as an internal control. All of the phenotypic assays were performed with three technical replicates and three independent biological repetitions with the same results. The primers that were used in this assay are listed in Table S1.

### Apoptosis rate measurement.

Different plasmids containing SUD2_core_ and Nsp5 were transfected into NHBE cells. Trypsin-digested cells (≈1 × 10^5^) cells were washed with PBS 3 times and were centrifuged for 1 min, at 2,000 × *g*. The pelleted cells were resuspended with staining buffer (4 μL Annexin V-enhanced GFP and 4 μL PI; CellorLab catalog number CX005L) and were incubated at room temperature for 10 min without light. The apoptosis rates of different cells were then immediately detected by flow cytometry, which showed green fluorescence in apoptotic cells, red and green fluorescence in dead cells, and almost no fluorescence in living cells.

### Cell proliferation calculation.

Cell suspensions were inoculated into 96-well plates. The culture plates were placed in an incubator for preculture for 24 h. Ten-microliter aliquots of CCK-8 solutions were added to each well and incubated for 1 h. The absorbance at 450 nm was measured with a microplate reader (Victor Nivo Alpha S, PerkinElmer, USA). The intervals of testing were 0, 1, 2, 3, 4, and 5 days.

### Quantification and statistical analysis.

The number of experiments and replicates are indicated in individual figure legends. Data were processed and visualized using Origin2021. All quantified data are represented as means ± standard errors of the means (SEM), as indicated, and quantification details are available in the figure legends. Western blotting band intensities were quantified using Image J.

### Data availability.

The data reported in this paper will be shared by the corresponding author upon request. Any source file required to reanalyze the AlphaFold modeling structure reported in this paper is available from the corresponding author upon request.
