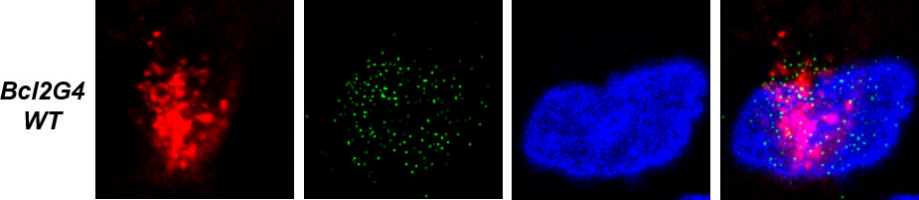# Correction for Li et al., “SARS-CoV-2 SUD2 and Nsp5 Conspire to Boost Apoptosis of Respiratory Epithelial Cells via an Augmented Interaction with the G-Quadruplex of BclII”

**DOI:** 10.1128/mbio.03871-24

**Published:** 2025-01-16

**Authors:** Ying Li, Quanwei Yu, Ridong Huang, Hai Chen, Hequan Ren, Lingling Ma, Yang He, Weimin Li

## AUTHOR CORRECTION

Volume 14, no. 2, e03359-22, 2023, https://doi.org/10.1128/mbio.03359-22. During preparation of the GFP image in the middle row of Fig. 2E, the GFP image from Fig. 2D was incorrectly used again. We have revised the image in Fig. 2E. The current values are correct, and the conclusions remain intact. We apologize for this error.

Page 6, Fig. 2E: The middle row should appear as in this correction.

**Fig 2:**