# Supplementary figures and images for: SARS-CoV-2 SUD2 and Nsp5 Conspire to Boost Apoptosis of Respiratory Epithelial Cells via an Augmented Interaction with the G-Quadruplex of BclII
Source: mBio. 2023 Feb 28;14(2):e03359-22. doi: 10.1128/mbio.03359-22 (PMC10127692; doi:10.1128/mbio.03359-22)

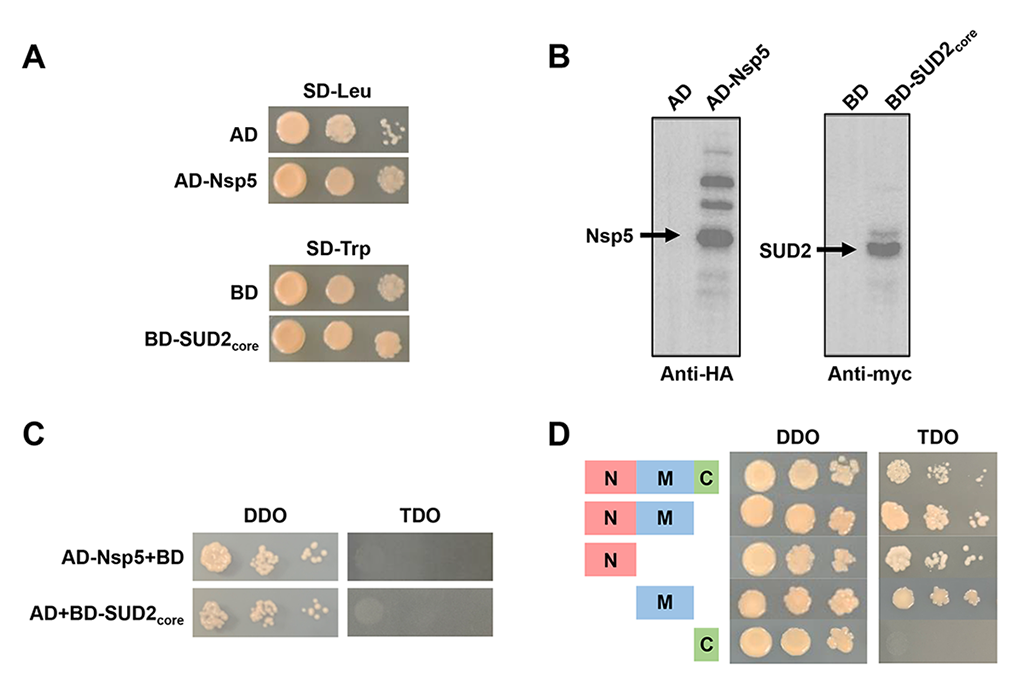

Supplement: FIG S1 [file mbio.03359-22-s0001.tif]

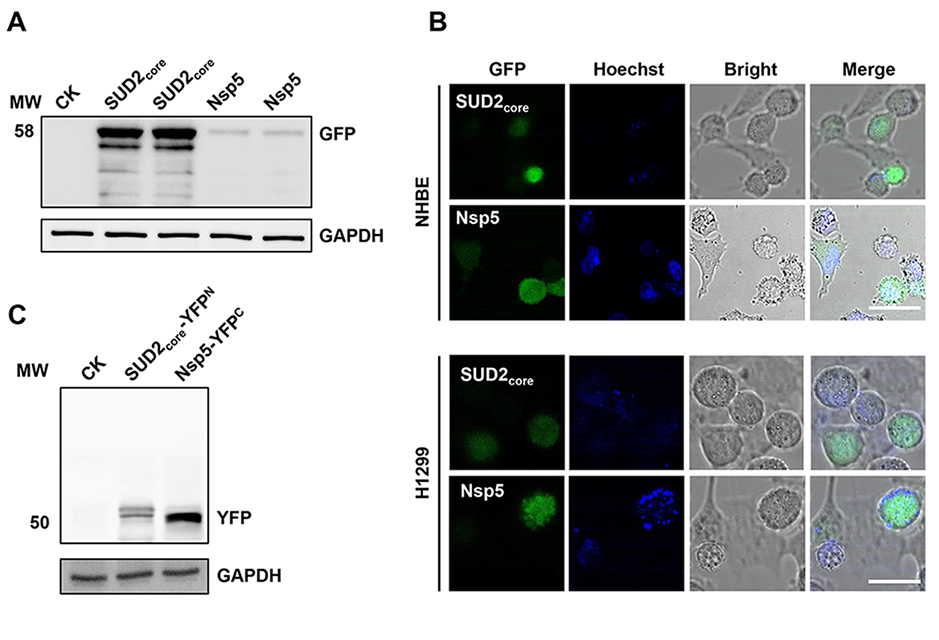

Supplement: FIG S2 [file mbio.03359-22-s0002.tif]

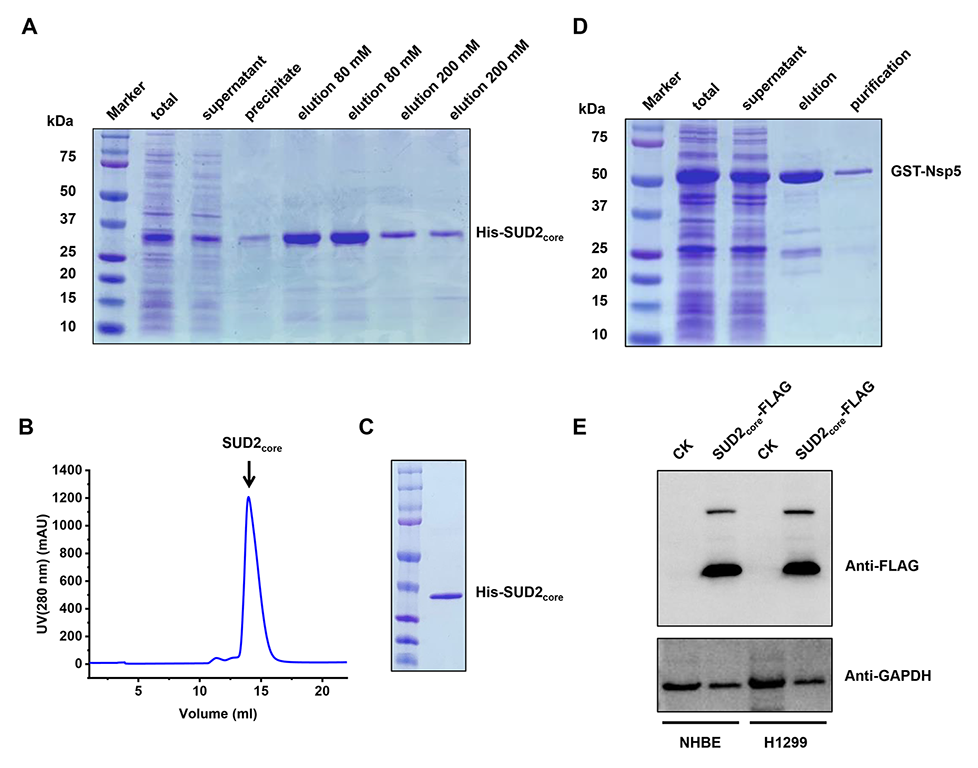

Supplement: FIG S3 [file mbio.03359-22-s0003.tif]

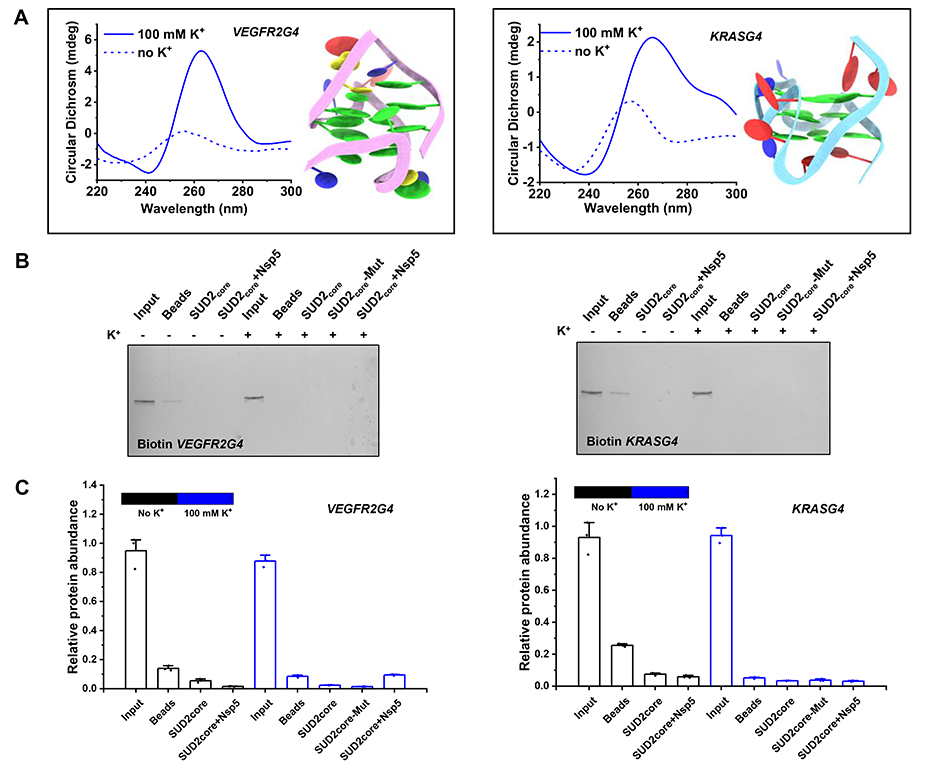

Supplement: FIG S4 [file mbio.03359-22-s0004.tif]

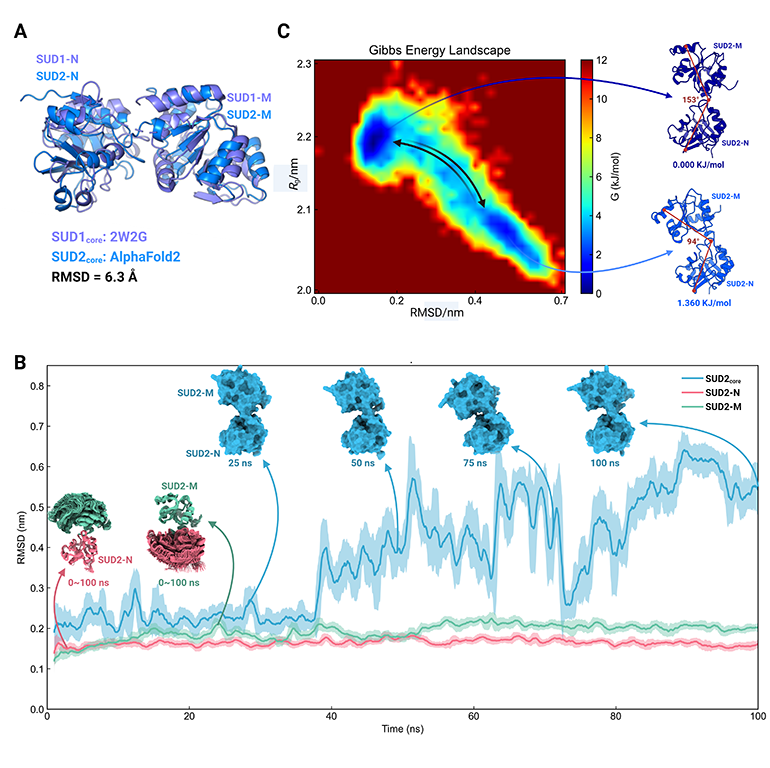

Supplement: FIG S5 [file mbio.03359-22-s0005.tif]

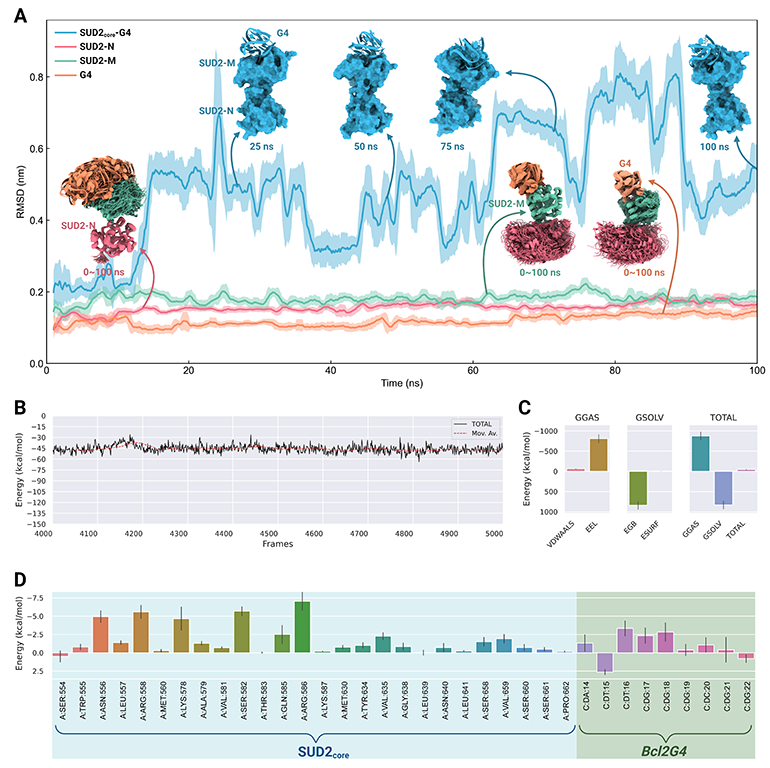

Supplement: FIG S6 [file mbio.03359-22-s0006.tif]

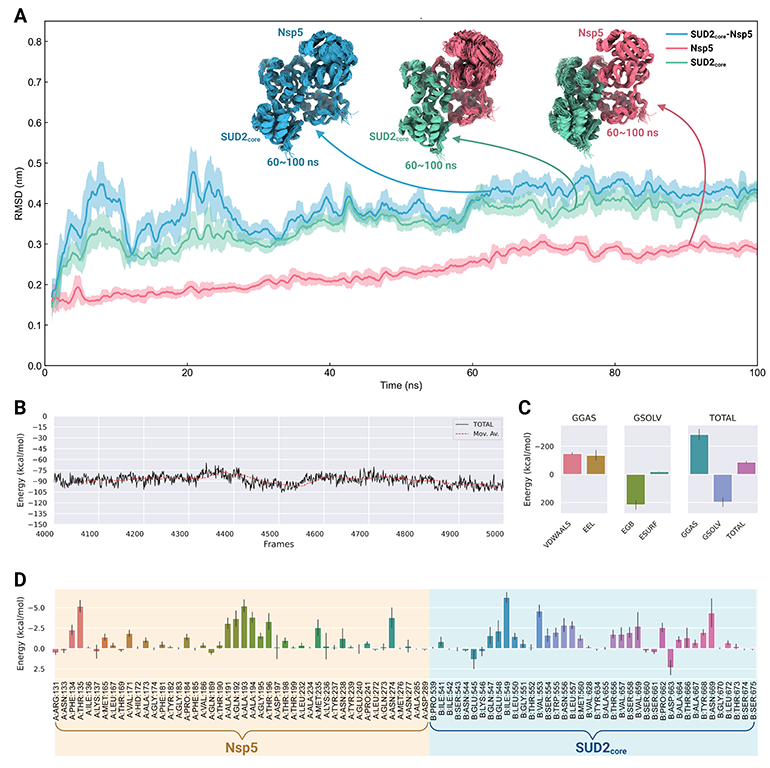

Supplement: FIG S7 [file mbio.03359-22-s0007.tif]

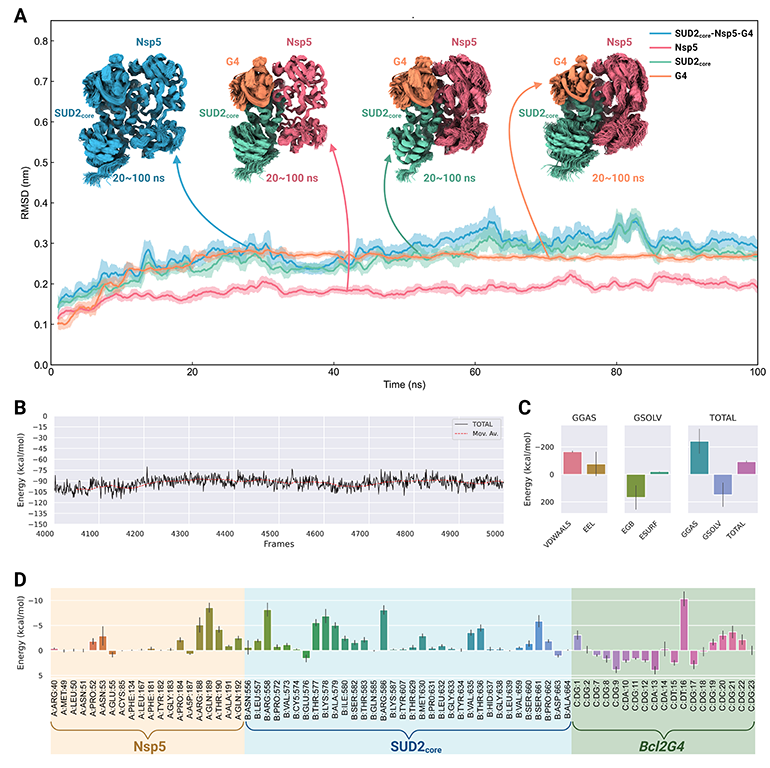

Supplement: FIG S8 [file mbio.03359-22-s0008.tif]

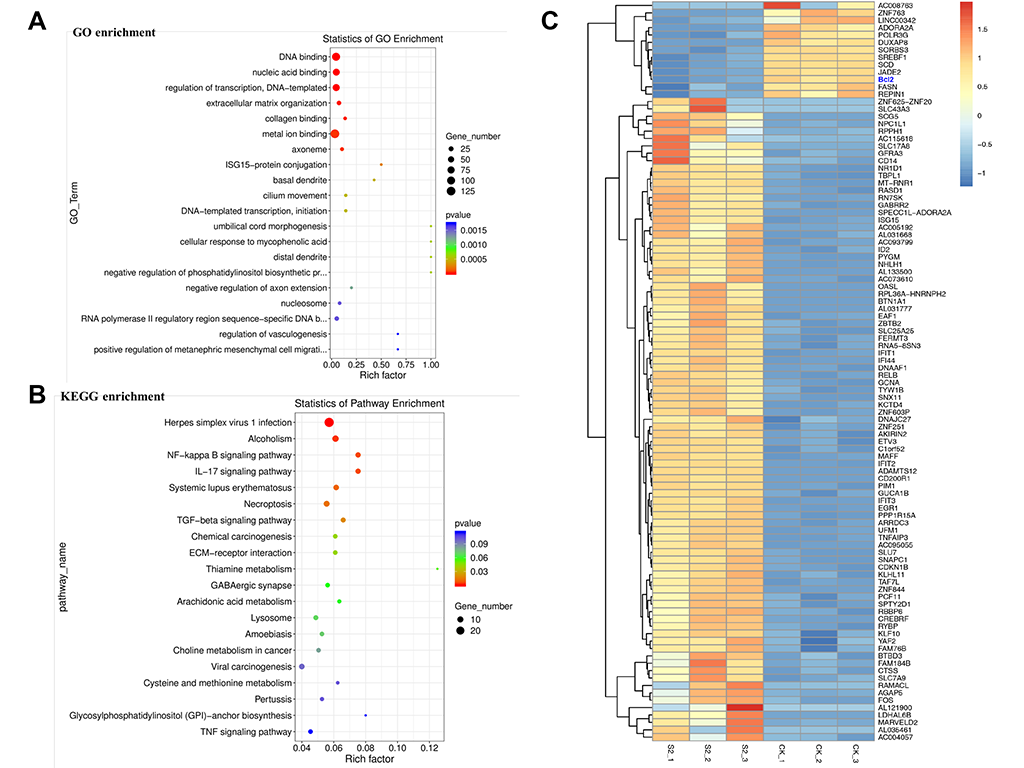

Supplement: FIG S9 [file mbio.03359-22-s0009.tif]
