# Supplementary material for: SARS-CoV-2 SUD2 and Nsp5 Conspire to Boost Apoptosis of Respiratory Epithelial Cells via an Augmented Interaction with the G-Quadruplex of BclII
Source: mBio. 2023 Feb 28;14(2):e03359-22. doi: 10.1128/mbio.03359-22 (PMC10127692; doi:10.1128/mbio.03359-22)
Supplement: TABLE S1 [file mbio.03359-22-s0010.docx]

| Y2H assay |  |
| --- | --- |
| SUD2-Full-F | CGGAATTCATGAAAATCAAAGCTTGTGTTGAAGAAG |
| SUD2-Full-R | CGGGATCCAGAAAGAAGTGTCTTAAGATTGTCA |
| SUD2-N+M-F | CGGAATTCATGAAAATCAAAGCTTGTGTTGAAGAAG |
| SUD2-N+M-R | CGGGATCCAGAAGAAGTAAGATAACCATTATAC |
| SUD2-N-F | CACGGAATTCATGAAAATCAAAGCTTGTGTTGAAGAAG |
| SUD2-N-R | CGGGATCCTTCTTGCTTCTCATTAGAGATAATA |
| SUD2-M-F | CGGAATTCATGGGAACTGTTTCTTGGAATTTGCGAG |
| SUD2-M-R | CCGGGATCCAGAAGAAGTAAGATAACCATTATAC |
| SUD2-C-F | CGGAATTCATGGAAGAACATTTTATTGAAACCATCT |
| SUD2-C-R | CGGGATCCAGAAAGAAGTGTCTTAAGATTGTCA |
| Nsp5-F | CGGAATTCATGAGTGGTTTTAGAAAAATGGCA |
| Nsp5-R | CGGGATCCTTGGAAAGTAACACCTGAGCAT |
| Subcellular localization |  |
| SUD2-GFP-F | CGGGATCCATGAAGAAAATCAAAGCTTGTGTTGAAG |
| SUD2-GFP-R | CGGAATTCAGAAGAAGAAGTAAGATAACCATTA |
| Nsp5-GFP-F | CCGGATCCATGAGTGGTTTTAGAAAAATGGCATTCC |
| Nsp5-GFP-R | CGGAATTC TTGGAAAGTAACACCTGAGCATTGT |
| BiFC assay |  |
| SUD2-VN-F | CGGAATTCCGATGAAGAAAATCAAAGCTTGTGTTGAAG |
| SUD2-VN-R | CTAGTCTAGATAGAAGAAGAAGTAAGATAACCATTA |
| Nsp5-VC-F | CGGAATTCCGATGAGTGGTTTTAGAAAAATGGCATTCC |
| Nsp5-VC-R | CCGCTCGAGTTTGGAAAGTAACACCTGAGCATTGT |
| Protein expression |  |
| SUD2-His-F | CGGAATTCATGAAGAAAATCAAAGCTTGTGTTGAAG |
| SUD2-His-R | ACGCGTCGACAGAAGAAGAAGTAAGATAACCATTA |
| Nsp5-GST-F | CGGGATCCATGAGTGGTTTTAGAAAAATGGCATT |
| Nsp5-GST-R | CGGAATTCTTGGAAAGTAACACCTGAGCATTG |
| Luciferase activity assay |  |
| pBcl2-F | GGTTGTGCCTGGAATTGTTTATAATGTG |
| pBcl2-R | AAGAGATTTATTTCCAATAAAATTTAAATTTTATACT |
| q-PCR assay |  |
| Bcl2-F | CCTGTGGATGACTGAGTACCTG |
| Bcl2-R | AGCCAGGAGAAATCAAACAGAGG |
| Apaf1-F | AACCAGGATGGGTCACCATA |
| Apaf1-R | ACTGAAACCCAATGCACTCC |
| Bak1-F | TGGGCTTCGGCTACCGTCTGGC |
| Bak1-R | GTTCAGGATGGGACCATTGC |
| Caspase3-F | TCGGTCTGGTACAGATGTCG |
| Caspase3-R | GGCTCAGAAGCACACAAACA |
| Caspase8-F | GGTTAGGGGACTCGGAGACT |
| Caspase8-R | CAGGCTCAGGAACTTGAGGG |
| Caspase7-F | GTGGGAACGATGGCAGATGA |
| Caspase7-R | CGGGTGGTCTTGATGGATCG |
| Caspase9-F | AGGCCCCATATGATCGAGGA |
| Caspase9-R | TCGACAACTTTGCTGCTTGC |
| Akt2-F | TGCCACCATGAATGAGGTGAATA |
| Akt2-R | CAGCGTATGACAAAGGTGTTGG |
| GAPDH-F | ACAACTTTGGTATCGTGGAAGG |
| GAPDH-R | GCCATCACGCCACAGTTTC |
| G4-DNA |  |
| Bcl2G4WT | GGGCGCGGGAGGAATTGGGCGGG |
| Bcl2G4Mut | GAGCGCGAGAGGAATTGAGCGAG |
| VEGFRG4 | GGGTACCCGGGTGAGGTGCGGGGT |
| KRASG4 | AGGGCGGTGTGGGAATAGGGAA |
